# Mitophagy and Neurodegeneration: Between the Knowns and the Unknowns

**DOI:** 10.3389/fcell.2022.837337

**Published:** 2022-03-22

**Authors:** Cuckoo Teresa Jetto, Akshaya Nambiar, Ravi Manjithaya

**Affiliations:** ^1^ Autophagy Laboratory, Molecular Biology and Genetics Unit, Jawaharlal Nehru Centre for Advanced Scientific Research, Bengaluru, India; ^2^ Neuroscience Unit, Jawaharlal Nehru Centre for Advanced Scientific Research, Bengaluru, India

**Keywords:** mitophagy, mitochondrial dynamics, ubiquitination, phosphorylation, mitochondrial dysfunction, neurodegenaration

## Abstract

Macroautophagy (henceforth autophagy) an evolutionary conserved intracellular pathway, involves lysosomal degradation of damaged and superfluous cytosolic contents to maintain cellular homeostasis. While autophagy was initially perceived as a bulk degradation process, a surfeit of studies in the last 2 decades has revealed that it can also be selective in choosing intracellular constituents for degradation. In addition to the core autophagy machinery, these selective autophagy pathways comprise of distinct molecular players that are involved in the capture of specific cargoes. The diverse organelles that are degraded by selective autophagy pathways are endoplasmic reticulum (ERphagy), lysosomes (lysophagy), mitochondria (mitophagy), Golgi apparatus (Golgiphagy), peroxisomes (pexophagy) and nucleus (nucleophagy). Among these, the main focus of this review is on the selective autophagic pathway involved in mitochondrial turnover called mitophagy. The mitophagy pathway encompasses diverse mechanisms involving a complex interplay of a multitude of proteins that confers the selective recognition of damaged mitochondria and their targeting to degradation *via* autophagy. Mitophagy is triggered by cues that signal the mitochondrial damage such as disturbances in mitochondrial fission-fusion dynamics, mitochondrial membrane depolarisation, enhanced ROS production, mtDNA damage as well as developmental cues such as erythrocyte maturation, removal of paternal mitochondria, cardiomyocyte maturation and somatic cell reprogramming. As research on the mechanistic aspects of this complex pathway is progressing, emerging roles of new players such as the NIPSNAP proteins, Miro proteins and ER-Mitochondria contact sites (ERMES) are being explored. Although diverse aspects of this pathway are being investigated in depth, several outstanding questions such as distinct molecular players of basal mitophagy, selective dominance of a particular mitophagy adapter protein over the other in a given physiological condition, molecular mechanism of how specific disease mutations affect this pathway remain to be addressed. In this review, we aim to give an overview with special emphasis on molecular and signalling pathways of mitophagy and its dysregulation in neurodegenerative disorders.

## Introduction

Mitochondria are highly dynamic intracellular organelles that maintain cellular homeostasis by coordinating a myriad of functions such as ATP generation, regulation of cell death, maintenance of calcium homeostasis, lipid and carbohydrate metabolism, and intracellular signalling (Friedman and Nunnari 2014). As these unique organelles determine cell fate both in terms of survival and death, maintaining mitochondrial homeostasis is very critical. Owing to their crucial role in a cell, mitochondrial dysfunction is associated with many chronic disorders some of which are neurodegeneration, aging, cardiomyopathies and cancer (Murphy et al., 2016; Zong et al., 2016; Johnson et al., 2021). Therefore, cells have evolved stringent quality control mechanisms that function through a highly orchestrated hierarchy, such as fission-fusion dynamics (Shah et al., 2021), mitophagy (Onishi et al., 2021), mitochondrial unfolded protein response (mitoUPR) (Shpilka and Haynes 2018), and mitochondrial derived vesicles (MDV) (Sugiura et al., 2014) to maintain an optimal pool of healthy mitochondria. In mitophagy, which is a specialised form of selective macroautophagy, damaged mitochondria are engulfed into a double-membrane vesicles called mitophagosomes and are delivered to lysosomes for degradation. The pathway comprises of complex interplay of diverse proteins, which recognise the damaged mitochondria and selectively target them for degradation *via* mitophagy. Through extensive research in the past 2 decades, while intricate molecular details of this pathway have been unravelled, several unanswered questions particularly about the capture and delivery of damaged mitochondria to lysosomes remain to be elucidated. In this review, we are trying to focus on two major aspects regarding mitophagy pathway, firstly the mechanisms through which lesser explored molecular players aid in the completion of this pathway, and secondly, the molecular details of how dysregulation in this pathway contribute to pathological conditions such as neurodegenerative disorders.

## Molecular Players Governing Mitochondrial Organelle Dynamics and Mitophagy

### Mitochondrial Dynamics and Mitophagy

The morphological dynamics of the mitochondria are maintained by interconnected processes that control the fission-fusion dynamics enabling the organelle to respond to a plethora of cellular stresses by rapid changes in their structure and function. Anatomically, mitochondria form tubular structures that is divided into outer mitochondrial membrane (OMM), inner mitochondrial membrane (IMM), and intermembrane space (Kühlbrandt 2015). Each of these membrane surfaces are enriched with proteins that control its morphological dynamics. One of the cellular strategies to combat mitochondrial damage is to separate the damaged part of the mitochondrion from its healthy counterparts. This is achieved through mitochondrial fission, a process that can sever fragments of damaged mitochondria which act as substrates for mitophagy. Mitochondrial fusion on the other hand serves the purpose of establishing and maintaining reticulate structures of the organelle, wherein, at times, fusion of the impaired mitochondria with healthy ones dilutes the enormity of damage (Twig and Shirihai 2011). It is therefore believed that fusion opposes the onset of mitophagy. In this section, we aim to throw some light on the crosstalk between mitochondrial membrane dynamics and mitophagy mediated by different molecular players.

#### Mitochondrial Membrane Proteins

Fission of the mitochondrial network is primarily regulated by a member of the dynamin superfamily of GTPases, known as Dynamin Related Protein 1 (DRP1). DRP1 present in the cytosol is recruited to the membrane of the mitochondria, by the receptors present on the outer membrane surface where it self-oligomerises into complexes that enhance its hydrolytic activity (Losón et al., 2013). DRP1 assembles as a ring around the mitochondrial tubule mediating its fission (Smirnova et al., 2001). Mutations in DRP1 disrupts fission, enhancing the elongation of mitochondrial network (Banerjee et al., 2021). DRP1 null mutation in mice results in embryonic lethality with severe neural and developmental defects (Ishihara et al., 2009). In yeast, DRP1 homolog, Dnm1 (Mozdy et al., 2000) is recruited to the OMM with the aid of accessory proteins, such as, mitochondrial fission 1 protein (Fis1), CCR4-associated factor 4 (Caf4), and mitochondrial division protein 1 (Mdv1). In mammals, DRP1 is docked onto the mitochondrial membrane by Fis1, mitochondrial fission factor (Mff) (Gandre-Babbe and van der Bliek 2008; Liu and Chan 2015), and mitochondrial dynamics proteins of 49 kDa (MiD49), and 51 kDa (MiD51) (Palmer et al., 2011; Palmer et al., 2011). Loss of these receptors results in a reduction of DRP1 localisation on the mitochondrial surface, impairing fission cycles of the organelle (Losón et al., 2013). A recent study suggest the novel role of MiD51 protein in mitophagy where the depletion of this protein induces elongation of the mitochondrial network with enhanced Parkin-dependent clearance of damaged mitochondria, challenging the traditional convention that mitochondrial fission precedes mitophagy (Xian and Liou 2019). In another study, depletion of DRP1 along with Vacuolar Protein Sorting protein 13D (VPS13D) in neuronal cells, resulted in the accumulation of smaller mitochondrial fragments and larger mitophagy stalled intermediates. They also reported moonlighting functions of VPS13D in recruiting the downstream autophagic pathway proteins in engulfing the damaged mitochondria (Anding et al., 2018). Another fission-promoting protein Fis1, initially identified in yeast indirectly controls the mitochondrial fission rates by recruiting Dnm1 (DRP1 homologue in yeast cells) onto the OMM (Mozdy et al., 2000). Fis1 is reported to promote mitochondrial fission by interacting with the ER-mitochondria contact site proteins, elevating the calcium influx levels in a DRP1 independent manner (Iwasawa et al., 2011). It is also reported to competitively bind and inhibit the activity of fusion proteins, mitofusin1 (MFN1) and mitofusin2 (MFN2) (Yu-Jie Li et al., 2019). Although Fis1 is known to partake in mitochondrial fission, its absence is reported to be dispensable for the fission cycles, but studies from the *Caenorhabditis elegans* model system suggest its role in the clearance of damaged mitochondria on treatment with drugs that affect the function of mitochondria (Shen et al., 2014). It was perceived that fission is an important process that governs both mitochondrial biogenesis and clearance but a recent study reported differential localisation of mitochondrial proteins on its membrane surface that enabled scission and marked distinct fates of the mitochondrial fragments. While the mid-zone division, mediated by DRP1 and Mff led to mitochondrial biogenesis, the end-zone fission by DRP1 and Fis1 protein increased the contact of the mitochondrial fragments with lysosomes, thereby marking it for degradation (Kleele et al., 2021). This study also strengthens the hypothesis of Fis1 protein bridging mitochondrial fragmentation with mitophagy.

The fusion machinery is regulated by mitofusin proteins (MFN1 and MFN2) on the outer mitochondrial membrane and they also belong to a family of transmembrane GTPases. MFN1, owing to its higher GTPase activity regulates mitochondrial fusion and docking process much more efficiently than MFN2 (Escobar-Henriques and Joaquim 2019). MFN2 has received attention as an ER-mitochondria contact site tether protein and is reported to get ubiquitinated during PTEN induced kinase 1 (PINK1) - Parkin (PRKN) mediated mitophagy followed by p97 mediated proteasomal degradation. MFN2 degradation thus disrupts ER-mitochondria contacts promoting mitophagy (McLelland et al., 2018). It also recruits Parkin onto damaged mitochondria as a consequence of PINK1 dependent phosphorylation (Chen et al., 2021). IMM fusion is regulated by optic atrophy 1 (OPA1) which undergoes proteolytic cleavage by two peptidases namely Overlapping activity with m-AAA protease (OMA1) (Head et al., 2009), and YME1 like 1 (YME1L1) (Mishra et al., 2014) to produce long and short transmembrane forms, i.e., l-OPA1 and s-OPA1 respectively. l-OPA1, due to its low intrinsic hydrolytic activity works in concert with cardiolipin (mitochondria specific lipid) (Ban et al., 2018), and MFN1 (Song et al., 2009) to mediate fusion of IMM. Interestingly, higher levels of s-OPA1, present on the IMM, has been reported to inhibit fusion activity (Wang et al., 2021).

#### Cytoskeletal Elements

##### Actin and Myosin Motor Proteins

Filamentous actin (F-actin) plays a critical role in maintaining the morphological homeostasis of the organelle by aiding efficient docking of DRP1 protein onto the mitochondrial membrane at the time of fission (Hatch et al., 2016). DRP1 protein assembles on the OMM and constricts the membrane to mediate fission, but often, DRP1 oligomeric rings are smaller for constricting the entire circumference of the mitochondrial membrane, and hence a pre-constriction step is required. Pre-constriction of the mitochondrial membrane before fission is a coordinated step involving actin-associated proteins, F-actin and myosin motor proteins (Hatch et al., 2016). An isoform of actin nucleator protein formin, IFN2, is anchored on ER membranes and polymerizes actin, thus driving ER membranes closer to the mitochondria (Chakrabarti et al., 2018). This IFN2 mediated actin polymerization is aided by myosin motor protein, NMIIA (Kruppa and Buss 2021). Additionally, IFN2 also directly interacts with another protein Spire1C that is localized on the mitochondrial surface and promotes polymerization of actin filaments (Manor et al., 2015). IFN2-Spire1C interaction facilitates enwrapping of the ER tubules around mitochondria facilitating the decrease of membrane circumference (Friedman et al., 2011). Actin filaments on the mitochondria thus constrict the mitochondrial membrane surface which thereby enhances the docking of DRP1 protein onto the OMM, mediating fission. Also, DRP1 activity is enhanced in the presence of F-actin as shown by *in vitro* GTPase assay (Ji et al., 2015) suggesting that along with driving the constriction of the mitochondria, filamentous actin also primes DRP1 protein to form functional oligomers that assemble on the OMM. Treating the cells with fragmentation-inducing drugs such as carbonyl cyanide *m*-chlorophenylhydrazone (CCCP) trigger actin filaments to rapidly associate with mitochondrial fragments in an Actin Related Protein 2/3 (Arp2/3) dependent manner (Sunan Li et al., 2015). Another actin-associated protein, cofilin1, also plays an integral role in maintaining mitochondrial morphology and mitophagy. Knockdown of cofilin1 in mouse embryonic fibroblasts (MEFs) resulted in fragmented mitochondria with increased levels of DRP1 on OMM (Li et al., 2018). Also, it was observed that the mitochondrial morphology was restored in cofilin1-deficient MEFs upon expression of a constitutive active cofilin1 mutant, but not upon expression of a cofilin1 mutant that does not bind actin (Rehklau et al., 2017). These results support a model wherein cofilin dependent depolymerization of actin acts as a negative regulator of mitochondrial scission by antagonizing with IFN2-Spire1C mediated actin polymerization. In another report, cofilin1 was observed to depolymerize F-actin filaments post fission resulting in the disassembly of fission complex and reduction of mitochondrial membrane potential that triggered PINK1/Parkin dependent mitophagy (Li et al., 2018). Actin cages around the damaged mitochondria also serve as platforms for the growing phagophores, facilitating their capture (Kruppa and Buss 2018; Hsieh and Yang 2019). Additionally, Arp2/3 complex has also been implicated in mitophagosome biogenesis and blocking actin polymerisation using a Arp2/3 inhibitor led to the inhibition of mitophagosome formation by probably reducing surface accessibility for phagophore expansion (Hsieh and Yang 2019).

Together with actin, actin-associated motor proteins also play a vital role in mitochondrial membrane morphology, anchorage, docking and mitophagy. Myosin VI (MYO6), a unique unconventional myosin, has binding sites for ubiquitin and different autophagy adaptor proteins (de Jonge et al., 2019). It is targeted to the surface of damaged mitochondria in a Parkin-dependent manner, as a C431S mutation in the Parkin protein diminishes MYO6 translocation onto the mitochondrial surface (Kruppa et al., 2018). MYO6 thus forms a complex with Parkin and is recruited to the surface of damaged mitochondria *via* its ubiquitin binding ability. This recruitment enhances the assembly of actin cages around the damaged mitochondria resulting in their “bite-sized” fragments. While these fragments are unable to fuse back to the neighbouring healthy mitochondria, they promote the assembly of the downstream autophagic machinery leading to its engulfment (Kruppa and Buss 2021). MYO6 through its interaction with translocase of the outer membrane1 (TOM1) complex is also involved in mitophagosome maturation by facilitating its fusion to endosomes and subsequently to lysosomes (Tumbarello et al., 2012; Hu et al., 2019). Non-muscular myosins, NMIIA, NMIIB, and NMIIC, as previously discussed, accumulate at the site of mitochondrial fission and mediate actin-dependent constriction of the membrane (Korobova, Ramabhadran, and Higgs 2013; Hatch et al., 2016; Yang and Svitkina 2019; Ji et al., 2015) increasing assembly of DRP1 rings around the mitochondrial filaments. A pathogenic mutation in NMIIC, known to be associated with peripheral neuropathies, inhibited fission in a dominant-negative manner (Almutawa et al., 2019). NMIIB is reported to be involved in the pathogenesis of TAR DNA binding protein 43 (TDP-43) associated neurodegeneration where its knockdown led to mitochondrial accumulation with decreased neuronal cell viability (Jun et al., 2020). Inhibition of myosin II with blebbistatin or loss of NMIIA reduced mitochondrial fission and mitophagy in cardiomyocytes after ischemia–reperfusion injury (Korobova, Gauvin, and Higgs 2014; Kruppa and Buss 2021). Another unconventional myosin, myosin 19 (MYO19), has received a lot of attention in recent years with respect to mitophagy. MYO19 is a mitochondrial myosin that docks onto the surface *via* its interaction with Mitochondrial Rho (Miro) proteins (López-Doménech et al., 2018; Bocanegra et al., 2020) or directly through its tail domain. It is involved in the docking and anchorage of the organelle on actin filaments and proper segregation of the mitochondria during cytokinesis (Shneyer et al., 2016). Loss or overexpression of MYO19 results in the aggregation of the mitochondria near the perinuclear region indicating its role in the maintenance of mitochondrial morphology and dynamics (Oeding et al., 2018).

##### Microtubules, Kinesins and Dyneins

Mitochondrial trafficking majorly occurs along microtubules. Changes in the arrangement and post-translational modifications (PTM’s) in microtubules impact mitochondrial trafficking, positioning, and functionality inside a cell. A major chunk of the mitochondrial motility is coordinated by the microtubular tracks and hence any disturbances in its assembly are manifested as perturbations in fission-fusion dynamics (Ligon and Steward 2000). In *Saccharomyces pombe*, depolymerisation of the microtubules resulted in enhanced fragmentation of the mitochondria (Tianpeng Li et al., 2015; Mehta et al., 2019). On the contrary, in the cells of *Dictyostelium discoideum,* it led to a decrease in the rate of fission-fusion cycles (Woods et al., 2016). The proteins associated with the microtubules (MAPs) also modulate the organisation and assembly of the organelle. A microtubule stabilising protein Tau, when stably expressed, resulted in the perinuclear aggregation of mitochondria and reduced their transport to the cell periphery in chinese hamster ovary cells (CHO) and differentiated neuroblastoma N2a cells (Ebneth et al., 1998). In fission yeast, *Schizosaccharomyces pombe,* mitochondria were found to be associated with the microtubules by a linker protein, microtubule-mitochondria binding protein1 (Mmb1) and its absence resulted in the fragmentation of the mitochondria preceded by the dissociation of the organelle from the track. This study also shows that the physical association of the microtubular track with the mitochondria impedes its scission by inhibiting the assembly of the fission-related protein Dnm1 (DRP1 in mammals) onto the OMM (Mehta et al., 2019). Ubiquitously expressed neuronal homologue of microtubule-associated protein 1A and 1B, MAP1S is reported to inter-bridge three components, the microtubule, autophagic machinery by interacting with microtubule-associated protein 1A/1B-light chain 3 (LC3), and a Parkin interacting protein, leucine-rich PPR-motif containing protein (LRPPRC) on mitochondria, to initiate mitophagosomal biogenesis and transport (Xie et al., 2011). Mitochondria remain anchored at the axonal endings by a microtubule-binding protein syntaphilin (SNPH). Depletion of SNPH increased mitochondrial mobility and decreased its density at the axons (Kang et al., 2008). Release of SNPH from the damaged mitochondria enhanced their retrograde motility and decreased its PRKN mediated ubiquitination while overexpression of SNPH inhibited mitophagy in neurons (Mei-Yao Lin et al., 2017). Dynein carries the mitochondria in a retrograde fashion and has been immensely explored for its role in mitochondrial trafficking in neurons. Majority of dynein associated mutations affect the overall retrograde transport in the cell. In zebrafish, mutation affecting the function of dynactin subunit, actin related protein 11 (Arp11)/ACTR10 impaired the binding of dynein with the mitochondria (Drerup et al., 2017). Similarly, Dynein Cytoplasmic 1 Heavy Chain 1 (Dync1h1) mutation resulted in the accumulation of damaged mitochondria in the perinuclear region of fibroblasts (Eschbach et al., 2013). Defects in retrograde axonal transport of aged mitochondria by dynein have been observed to enhance its autophagic turnover at the axonal endings (Pilling et al., 2006). The retrograde transport induced by the binding of dynein motor protein to the mitochondria enhances mitophagy in neurons (Han et al., 2020). Kinesin-1 is the most documented motor protein driving the anterograde movement of mitochondria in neuronal and cardiac cells (Pilling et al., 2006). *In vitro* studies suggest a role of kinesin family member 1B (KIF1B) protein in mitochondrial transportation (Nangaku et al., 1994) and kinesin-like protein 6, KLP6, have been reported to regulate mitochondrial dynamics and movement (Tanaka et al., 2011). Trafficking Kinesin Proteins (TRAK1 and TRAK2), which are mammalian homologues of Milton in *Drosophila melanogaster*, act as motor adaptors linking dynein and kinesin motor proteins to coordinate both anterograde and retrograde movement of mitochondria (Fransson et al., 2003; Glater et al., 2006; Brickley and Stephenson 2011). While TRAK2 binds to dynein and hinders its association with kinesin-1 motor protein promoting mitochondrial trafficking in dendrites (van Spronsen et al., 2013; Loss and Stephenson 2015), TRAK1 binds to both dynein and kinesin motor proteins and mediates axonal transport of mitochondria (Glater et al., 2006). Over the past years, a growing body of evidence has dissected the role of mitochondrial Rho (MIRO) GTPase proteins in mitochondrial function, transport, and mitophagy. Miro proteins constitute a family of atypical GTPases and have two EF-hand motifs, two GTPase domains and a C- terminal tail domain that anchors the protein onto the OMM (Fransson et al., 2003; Klosowiak et al., 2016). Miro1/2 proteins interact with TRAK1/2 forming the Miro-TRAK complex that is involved in the coordination of mitochondrial motility on cytoskeletal tracks (Wang and Schwarz 2009; Schwarz 2013). Miro-mediated transport of mitochondria is also dependent on its acetylation status, where Histone deacetylase 6 (HDAC6) dependent acetylation at lys105 was observed to be critical for its motility (Kalinski et al., 2019).

**FIGURE 1:**
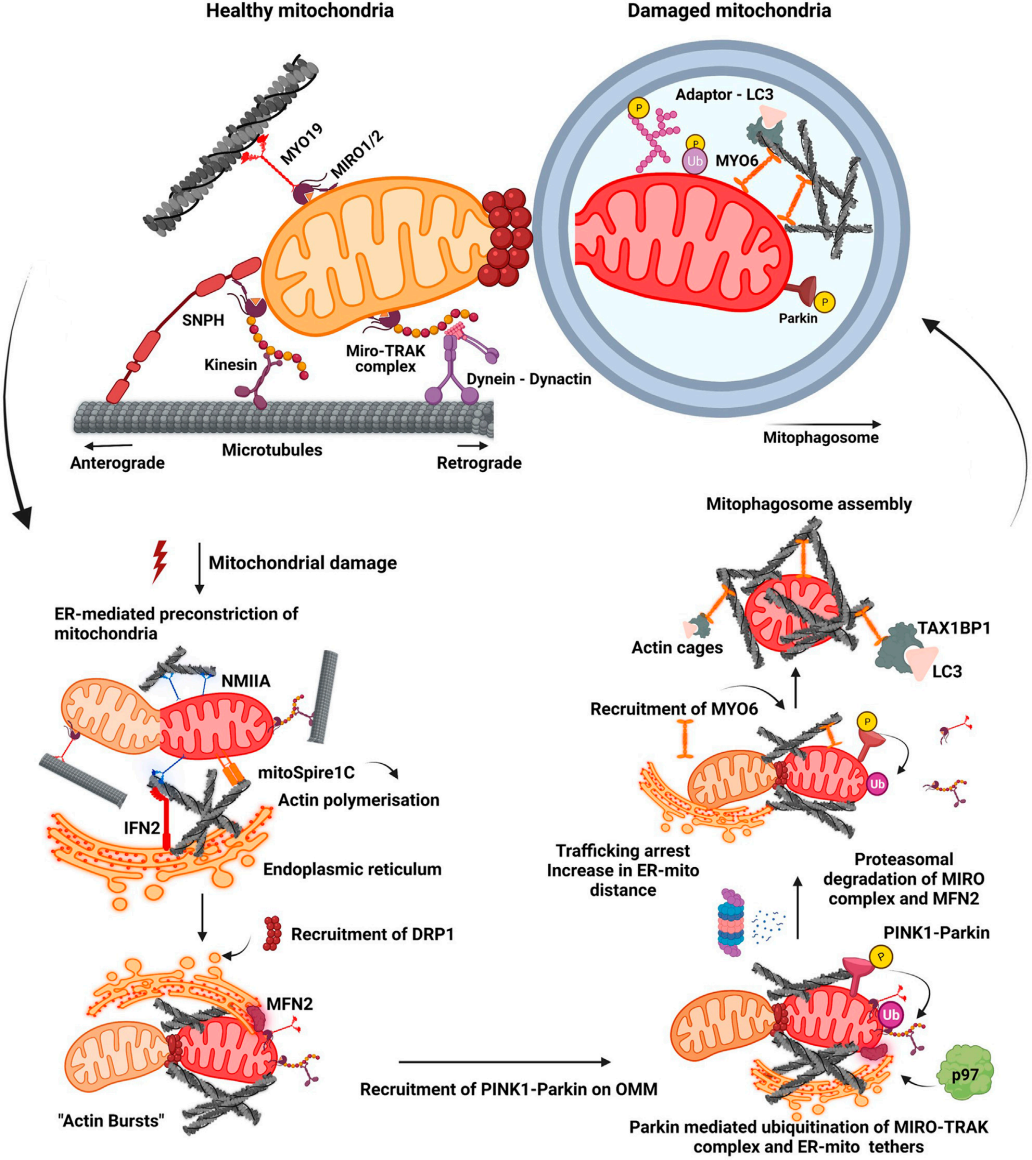
Mitochondrial fission-fusion dynamics and mitophagy. A healthy mitochondrion is endowed with proteins on its outer membrane such as SNPH, MYO19, dynein-dynactin complex, MIRO-TRAK complex, kinesins, which enable efficient trafficking of the organelle on cytoskeletal tracks. Onset of mitochondrial damage initiates constriction of mitochondrial membrane, wherein ER tightens around the mitochondrial filament mediated by IFN2, mitoSpire1C and NMIIA induced actin polymerisation. This pre-constriction step results in the recruitment of DRP1 onto the mitochondrial membrane. A burst of actin polymerisation around the damaged mitochondria is enhanced, resulting in its fragmentation from the healthy pool. Mitochondrial damage also leads to the localisation of PINK1-Parkin complex onto OMM, initiating a feed forward loop leading to proteasomal degradation of many OMM proteins such as MIRO proteins and MFN2. Degradation of these proteins results in the detachment of motor proteins and ER tethers. This process arrests trafficking of damaged mitochondria and also increases their distance from ER. Additionally, it also prevents the fusion of damaged mitochondria to the healthy pool. Actin filamentation now completely cages the damaged mitochondria, recruiting MYO6 which interacts with both ubiquitin and autophagy adaptor proteins. This whole cascade of events facilitates the engulfment of damaged mitochondria by autophagic machinery resulting in the formation of a mitophagosome which would eventually fuse with lysosome.

Mitophagy is triggered with the binding of PRKN to the OMM and subsequent phosphorylation by PINK1 leading to its enhanced activation, which in turn results in ubiquitination of mitochondrial proteins on OMM by PRKN. PINK1 has also been reported to phosphorylate Miro1/2 proteins which promotes PRKN-mediated ubiquitination of the Miro complex followed by its proteasomal degradation (Shlevkov et al., 2016; Safiulina et al., 2019). Phosphorylation of Miro proteins by PINK1 at S156, T298, and T299 positions serve as mitophagic signals, and each of these residues have been demonstrated to differentially regulate mitophagy (Wang et al., 2011). Phosphomimetic mutation at S156 recruits PRKN but is insufficient to drive mitophagy while phosphomimetic mutation at positions T298 and T299 inhibited PRKN mediated ubiquitination and thus mitophagy (Shlevkov et al., 2016). Loss of Miro proteins from OMM following its proteasomal degradation resulted in the shedding of MYO19 and kinesin motor proteins from OMM, arresting the motility of damaged mitochondria (Oeding et al., 2018; Pickles et al. 2018). This line of events arrests trafficking of damaged mitochondria enhancing their isolation and triggering the downstream events which are marked by “bursts of actin” filamentation-related processes around the damaged mitochondrion. This also culminates to enhance the local assembly of autophagic machinery resulting in clearance (Figure 1) (Kruppa and Buss 2021).

A recently reported process known as “Mitocytosis” characterised migrasome mediated removal of the damaged mitochondria. Authors have observed accumulation of damaged mitochondria inside migrasomes on inducing mitochondrial stress. According to the study, damaged mitochondria restricted their binding to dynein, hence inhibiting their retrograde movement and enhancing its peripheral localisation. Trafficking of damaged mitochondria towards the plasma membrane by kinesin family member 1B (KIF5B) and subsequent anchorage on the plasma membrane by MYO19 motor protein along with the fission protein complex orchestrated the efficient loading of damaged mitochondria into the migrasomes. As the cell underwent migration, migrasomes were shed off from the cell, thus also clearing the damaged mitochondria that hitchhiked in it (Jiao et al., 2021).

### Mitophagy Pathway

**FIGURE 2:**
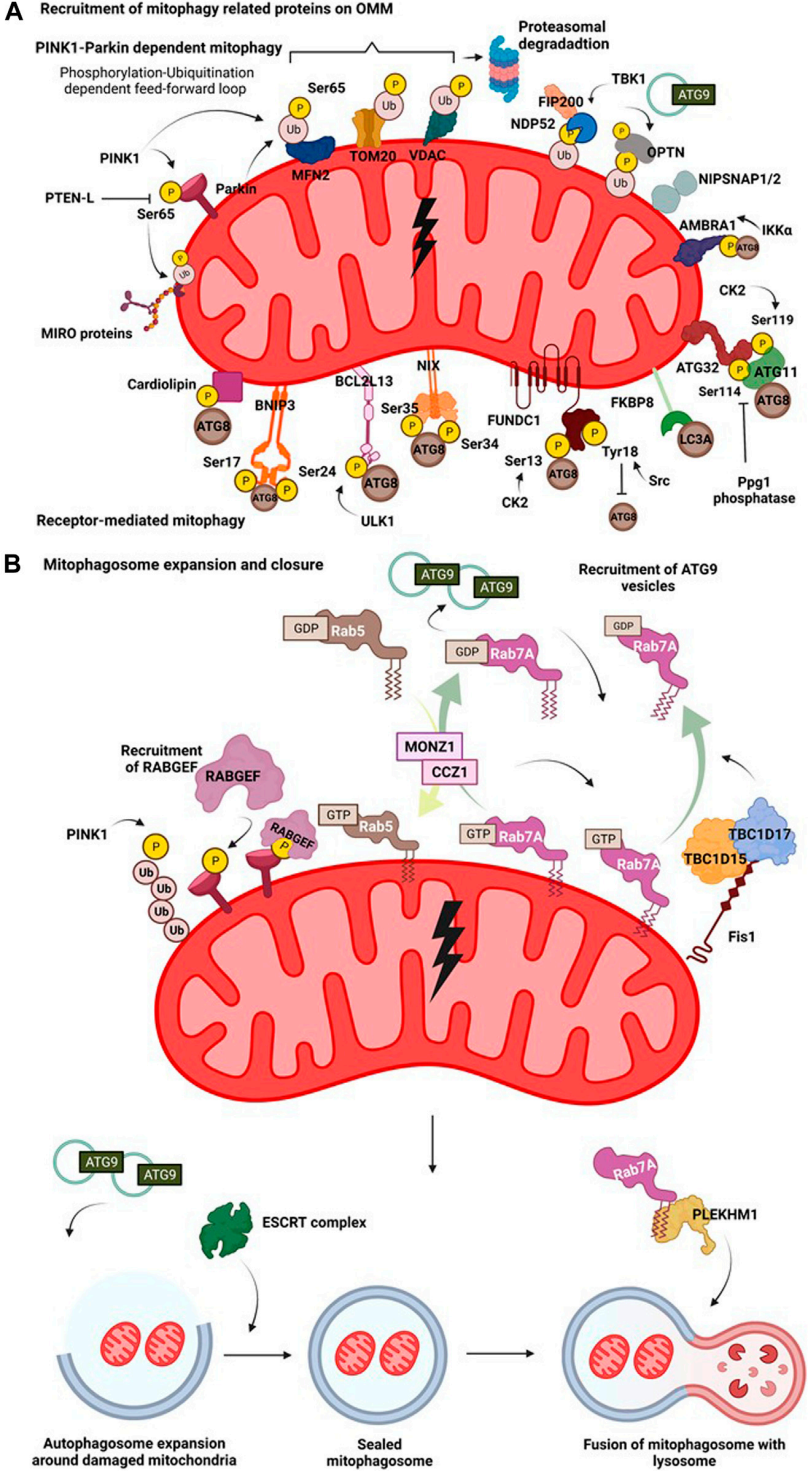
Mitophagy pathway. **(A)** **Recruitment of mitophagy related proteins on OMM**. On mitochondrial insult, the damaged part of the mitochondrial network undergoes autophagy mediated degradation. A damaged mitochondria recruits several proteins on their surface that interact with the autophagy machinery in receptor dependent or PINK1-Parkin dependent manner. Hypoxia induces recruitment of BNIP3 which upon phosphorylation at its Ser17 and Ser24 interacts with Atg8 family of proteins. Nix interacts with LC3 upon phosphorylation at its Ser34 and Ser35 positions. FUNDC1 interacts with the autophagy proteins and this is regulated by phosphorylation events at Ser13 by CK2 and Tyr18 by Src kinase. FKBP8 selectively interacts with LC3A. Mitochondrial damage results in phosphorylation of cardiolipin, that translocates from IMM to OMM and then interacts with autophagic machinery. AMBRA1 acts as a mitophagy receptor and is regulated by IKKα mediated phosphorylation. In yeast, binding of mitophagy receptor Atg32 with Atg11 and Atg8 is governed by phosphorylation at its Ser114 and Ser119 residues. Initial phosphorylation of Parkin by PINK1 at Ser65 in its ubiquitin domain triggers a feedforward loop recruiting more Parkin onto OMM. Parkin then ubiquitinates various OMM proteins such as MFN2, VDAC and Miro. This phosphorylation-ubiquitination mediated feed-forward loop amplifies the recruitment of different autophagy adaptor proteins such as NDP52 and OPTN. The phosphorylation of OPTN and NDP52 is mediated by TBK1. NIPSNAP proteins accumulate on the damaged mitochondria in a PINK1-Parkin dependent manner and recruit downstream autophagy proteins, promoting mitophagy. **(B)** **Mitophagosome expansion and closure**. Recruitment of mitophagy related proteins on OMM initiates an interaction of the proteins with components of autophagic machinery. The autophagosome then expands around the damaged mitochondria, engulfing them. This expansion is regulated by MONZ1-CCZ1 Rab GEF mediated Rab5 and Rab7 cycles. Phosphorylation Rab7 by TBK1 enhances its interaction with Atg9 vesicles. Mitochondrial GAPs, TBC1D15 and TBC1D17 functions downstream of Parkin and work in concert with FIS1 and LC3 to recruit Rab7 onto the mitochondrial surface shaping mitophagosome. Atg9 and Rab cycles mediate expansion of mitophagosome. Subsequently, the closure of mitophagosome is mediated by the ESCRT complex. Completely sealed mitophagosome then fuses with the lysosome facilitated by fusion proteins such as, PLEKHM1.

Mitophagy is a complex process wherein several protein complexes are recruited onto the damaged mitochondrion in a hierarchical manner targeting it for lysosomal degradation mediated by the autophagic machinery. Different mitochondrial stress signals such as hypoxia, membrane depolarisation, ROS accumulation, mtDNA damage, activate different pathways that flag the damaged mitochondria, eventually resulting in their elimination through autophagy pathway. Activation of these diverse pathways occurs as a result of PINK1-Parkin dependent ubiquitination of mitochondrial membrane proteins, ubiquitination independent receptor mediated pathways (Figure 2A), or Syntaxin17 (STX17) dependent mitophagy pathway.

#### Receptor-Mediated Mitophagy

In receptor-mediated mitophagy, mitochondrial damage triggers interaction of mitochondrial membrane proteins with Atg8 family of proteins *via* their LC3 interacting regions (LIR motifs), bringing autophagic machinery to the flagged site. Mitophagy receptors like Atg32, BNIP3, BCL2L13, NIX, FKBP8, FUNDC1, cardiolipin **(** Figure 2A **)**, remain inactive under steady-state levels and are activated by different PTMs only on specific cues that trigger the mitochondrial damage. Mitophagy in the budding yeast *Saccharomyces cerevisiae* is induced when yeast cells are grown till their stationary phase, or grown on non-fermentable carbon sources, or subjected to nitrogen starvation (Kanki and Klionsky 2010; Aoki et al., 2011). Through two different gene deletion screens, Atg32 was identified as the key receptor for mitophagy as its deletion was reported to abrogate the mitophagy pathway (Okamoto et al., 2009). On induction of mitophagy, Atg32 tends to accumulate on the OMM and is regulated by phosphorylation in its N-terminal domain at serine residues 114 and 119. Phosphorylation of Atg32 governs its interaction with Atg11 and Atg8 (Kanki et al., 2013; Aoki et al., 2011). Mutations at phospho-sites tend to disrupt the interaction between Atg32 and Atg11 and thus, Atg32-Atg11 axis is considered vital for bringing the components of autophagy machinery in close proximity, facilitating the process of mitophagy in yeast cells (Aoki et al., 2011; Kanki et al., 2013). Interestingly, functional homolog of Atg32, Bcl2-like protein 13 (BCL2L13), an OMM protein is also involved in maintaining the dynamicity of the organelle (Murakawa et al., 2015). In mammalian cells, BCL2L13 harbours two LIR regions and also interacts with the Unc-51 like autophagy activating kinase (ULK1) complex, and therefore might be involved in recruiting different players of the autophagy pathway to the site of mitochondrial damage (Murakawa et al., 2015). The expression level of Bcl2/adenovirus E1B 19 kDa protein-interacting protein 3 (BNIP3), an adapter for mitophagy, is transcriptionally upregulated under hypoxic conditions (Sowter et al., 2001; Chinnadurai, Vijayalingam, and Gibson 2008). While BNIP3 remains as an inactive monomer in the cytoplasm, following the onset of mitochondrial stress, it forms a stable homodimer anchoring itself on the OMM *via* its C-terminal domain (Chinnadurai, Vijayalingam, and Gibson 2008). Phosphorylation of BNIP3 at Ser17 and Ser24, adjacent to its LIR region promotes its binding to LC3 and mutations in its LIR motif have been reported to reduce the mitophagic activity inside cells (Zhu et al., 2013). NIX (also known as BNIP3L), another mitophagy receptor, is also induced on hypoxic stress and is involved in the removal of mitochondria from reticulocytes during its maturation. It shows high sequence similarity to BNIP3 (Sowter et al., 2001) and undergoes homodimerization on phosphorylation at Ser212 in its C-terminal end that enhances the localisation of autophagic machinery onto damaged mitochondria (Marinković et al., 2021). NIX can preferentially bind to LC3A, LC3B, Gamma-aminobutyric acid receptor-associated protein (GABARAP), Gamma-aminobutyric acid receptor-associated protein-like 1 and 2 (GABARAP-L1 and GABARAP-L2), and phosphorylation at Ser34 and Ser35 near its LIR motif has been shown to enhance its association with Atg8 family of proteins (Rogov et al., 2017). During ROS induced mitophagy, NIX also has been shown to associate in a complex that contains Rheb (Ras homolog enriched in brain, a small GTPase protein) and LC3 (Melser et al., 2013). Fun14 domain containing 1 (FUNDC1), is another hypoxia-regulated OMM receptor that is involved in mitophagy. The functionality of FUNDC1 as a mitophagy receptor under hypoxic conditions is regulated by phosphorylation-dephosphorylation events at positions Ser13 and Tyr18, near its N-terminally located LIR motif (Liu et al., 2012). Ser13 and Tyr18 are phosphorylated by casein kinase 2 (CK2) and Src tyrosine kinases respectively. Phosphorylation of Tyr18 by Src tyrosine kinase negatively regulates its interaction with LC3. But under hypoxic conditions, Src gets inactivated, resulting in reduced Tyr18 phosphorylation and relieving the negative regulatory effect on mitophagy axis (Liu et al., 2012). On mitochondrial depolarisation (CCCP treatment), FK506-binding protein 8 (FKBP8) through its N-terminally located LIR motif specifically interacts with LC3A, promoting autophagy-mediated degradation of mitochondria (Bhujabal et al., 2017) (Figure 2A). A recent study by employing proximity labelling based autophagosomal profiling had identified a novel p62-LC3C dependent piecemeal mitophagy pathway wherein LC3C was reported to mediate lysosomal degradation of metaxin 1 (MTX1), and this pathway is integral for maintaining mitochondrial dynamics at basal conditions. While the mechanism of interaction of MTX1 with LC3C is not clear, the presence of a non-canonical LIR motif in MTX1 and its involvement with p62 in this pathway requires further experimentation (Le Guerroué et al., 2017). In neuronal cells, upon mitochondrial depolarisation, the phospholipid cardiolipin, present on IMM, is rerouted to OMM. The cardiolipin present in OMM interacts with LC3 through LIR, thus recruiting the autophagic machinery, resulting in the engulfment of depolarised mitochondria (Chu et al., 2013).

#### PINK1-Parkin Dependent Mitophagy

PINK1-Parkin dependent mitophagy is the most well dissected out pathway in the field of mitophagy. PINK1, under basal conditions translocates to the mitochondria where it gets imported into the intermembrane space and undergoes degradation mediated by the proteasome and proteases (Greene et al., 2012). Upon mitochondrial insult, the import of PINK1 is prevented leading to its accumulation on OMM (Narendra et al., 2008). Subsequently, PINK1 phosphorylates Parkin, an E3 ubiquitin ligase of mitochondria at the Ser65 position in its ubiquitin-like domain (UBL) (Kondapalli et al., 2012). This phosphorylation initiates a feed forward amplification loop wherein more Parkin is recruited onto the OMM (Ordureau et al., 2014). Apart from directly phosphorylating Parkin, PINK1 also phosphorylates Ub chains present on the OMM at Ser65, which further serves as a platform for Parkin recruitment through direct binding (Kane et al., 2014; Okatsu et al., 2018). The binding of Ub-pSer65 to the ring finger protein 1 (RING1) domain of Parkin brings about a conformation change resulting in its activation (Wauer et al., 2015; Tang et al., 2017). It has also been reported that pSer65 Ub chains are resistant to the activity of deubiquitinating enzymes (DUBs) which in turn prolongs their lifespan on OMM and upregulates the process of mitophagy (Huguenin-Dezot et al., 2016; Gersch et al., 2017; Sato et al., 2017). Activated Parkin ubiquitinates different proteins on the surface such as MFN2 (Tanaka et al., 2010), translocase of outer membrane 20 (TOM20) (Yoshii et al., 2011), voltage-dependent anion channel (VDAC) (Ham et al., 2020) and Miro (Liang et al., 2015) directing them to proteasomal degradation (Figure 2A). Phosphorylation by PINK1 and subsequent ubiquitination by Parkin paves way for autophagy adaptor proteins and related machinery (discussed in the upcoming sections) to initiate the autophagic engulfment process. Several genetic screens have provided insights into the regulation of PINK1-Parkin activity. A pooled genome-wide CRISPR/Cas9 knockout screen identified THAP domain containing 11 (THAP11) as a transcriptional repressor negatively regulating abundance of Parkin and pSer65Ub on mitophagic induction (Potting et al., 2018). Another multidimensional CRISPR–Cas9 genetic screen identified adenine nucleotide translocator (ANT) complex as a regulator of PINK1 stabilisation on OMM during mitophagy. ANT complex was shown to have inhibitory effects on the pre-sequence of translocase of inner mitochondrial membrane 23 (TIM23) that relays to stabilise PINK1 on OMM (Hoshino et al., 2019). A siRNA screen combined with high-content microscopy led to the identification of translocase of outer mitochondrial membrane (TOMM7), Heat Shock Protein Family A (Hsp70) Member 1 Like (HSPA1L), BAG Cochaperone 4 (BAG4), Siah E3 Ubiquitin Protein Ligase Family Member 3 (SIAH3) as upstream regulators of PINK1-Parkin translocation on OMM following mitochondrial insult (Hasson et al., 2013). Sterol regulatory element binding transcription factor 1 (SREBF1), F-box and WD40 domain protein 7 (FBXW7) and ATPase inhibitory factor 1 (ATPIF1) were identified as positive regulators of Parkin translocation during mitophagy from a genome-wide RNAi screen (Lefebvre et al., 2013; Ivatt and Whitworth 2014). In another study, following mass spectrometric analysis of Autophagy-Linked FYVE (ALFY) and p62 interactors, two related proteins, NIPSNAP1 and NIPSNAP2 (4-Nitrophenylphosphatase domain and non-neuronal SNAP25-like protein homologs), were shown to accumulate on mitochondrial membrane upon depolarisation and was dependent on PINK1-Parkin localisation. They appear to have redundant roles in mitophagy pathway as only double knock-out of NIPSNAP1 and NIPSNAP2 in HeLa cells seem to abrogate PINK1-Parkin dependent mitophagy. On accumulating on damaged mitochondria, they act as “eat-me” signals, further aiding in the recruitment of Atg8 family of proteins. Interestingly, knockout of NIPSNAP1 affected mitophagy and resulted in Parkinsonism in a zebrafish model (Abudu et al., 2019).

#### STX17 Mediated Mitophagy

Molecular players of the mitophagy pathway have been dissected out by the application of different mitochondrial stress signals such as membrane depolarisation, ROS accumulation, hypoxic conditions, and mtDNA damage (Williams and Ding 2018). Recent evidence suggesting induction of mitophagy through the STX17-Fis1 axis represents one such pathway wherein mitophagy is observed without any ectopic stress signals. OMM protein, Fis1, maintains the mitochondrial dynamics and also prevents the translocation of STX17 from ER and mitochondria-associated ER membranes (MAM) onto mitochondria (Xian et al., 2019). Loss of Fis1 resulted in the translocation of STX17 onto the mitochondrial surface wherein it further signalled the hierarchical localisation of other proteins of the macro-autophagic machinery such as ATG14, WD repeat domain phosphoinositide-interacting protein 2 (WIPI2), zinc finger FYVE-type containing 1 (ZFYVE1/DFCP1) to promote autophagy-mediated engulfment of the mitochondria (Xian et al., 2019).

### Mitophagy Adaptors

Autophagy adaptors act as a bridge, linking the cargo destined for degradation with autophagy-related proteins. Adaptor proteins bind to the ubiquitinated cargo through their ubiquitin-binding domains and also engage with Atg8 family of proteins *via* their LIR motifs. Simultaneous genetic knockout of five autophagy adaptor proteins, p62, Tax1-binding protein 1 (TAX1BP1), Nuclear Dot Protein 52 kDa (NDP52), Neighbor of BRCA1 gene 1 (NBR1), and Optineurin (OPTN) by Richard Youle’s group revealed insights into the contribution of each adaptor in the mitophagy pathway. Through this study, it was reported that PINK1 mediated phosphorylation of Ubiquitin at Ser65 results in the recruitment of autophagic adaptors, OPTN and NDP52, independent of Parkin (Lazarou et al., 2015; Padman et al., 2019). While the role of adaptor proteins p62 and NBR1 was reported to be dispensable, OPTN, and NDP52 act as upstream receptors in the mitophagy pathway (Lazarou et al., 2015). The recruitment of OPTN onto damaged mitochondria and its binding with poly-Ub chains is regulated by phosphorylation at three sites, S177 position adjacent to its LIR motif, S473, and S513 position in its ubiquitin binding in ABIN and NEMO (UBAN) domain by TANK binding kinase 1 (TBK1) (Heo et al., 2015). NDP52 and OPTN have also been reported to directly engage with autophagy machinery proteins such as focal adhesion kinase family interacting protein of 200 kD (FIP200) (Vargas et al., 2019; Zhou et al., 2021), and ATG9A vesicles respectively (Yamano et al., 2020), thereby initiating the process of mitophagy. Spatio-temporal imaging in neuronal cells showed that on mitochondrial insult, fragmented mitochondria are encapsulated by OPTN, followed by LC3 recruitment onto them, but acidification of mitophagosomes is a comparatively slower process and thus forms a rate-limiting step in neuronal mitophagy (Evans and Holzbaur 2020) (Figure 2A).

### Mitophagosome Elongation and Closure

The molecular players involved in regulating autophagosomal expansion around damaged mitochondria is different as compared to the canonical autophagy pathway. Endosomal Rab proteins, Rab associated proteins and proteins of the ESCRT machinery have been studied for their role in mitophagosome expansion and closure. Two of the mitochondrial GTPase activating proteins (GAPs), TBC1 domain family member 15 (TBC1D15) and TBC1 domain family member 17 (TBC1D17) functioning downstream of Parkin activation, work in concert with Fis1 and LC3 to recruit Rab7 onto the mitochondrial surface shaping mitophagosome during mitophagy (Yamano et al., 2014). It was observed that absence of TBC1D15 results in the accumulation of elongated mitophagosomes in cells, suggestive of its role in mitophagosome expansion (Yamano et al., 2014; Jimenez-Orgaz et al., 2018). Another CRISPR/Cas9 screen also identified TBC1D15 as a part of the retromer complex acting upstream of Rab7. TBC1D15 was also observed to help in assembling of ATG9A tagged vesicles around the damaged mitochondria facilitating mitophagy (Jimenez-Orgaz et al., 2018). Additionally, phosphorylation of Rab7A at Ser72 promotes the recruitment of ATG9A vesicles around the damaged mitochondria (Heo et al., 2018). Guanine nucleotide exchange factors (GEFs) of Rab7A, monensin sensitivity protein 1 (MON1) and caffeine, calcium, and zinc 1 (CCZ1) act upstream to Rab7 localisation and govern the assembly of Rab7A onto damaged mitochondria in a Parkin dependent manner (Nordmann et al., 2010). A recent study using proximity-biotinylation approach, identified C5orf51, a component of MON1-CCZ1 complex as a positive regulator of Rab7A translocation to mitochondria upon membrane depolarisation. C5orf51 was also identified in a GWAS study pertaining to Alzheimer’s disease, therefore highlighting the potential role of C5orf51 in Rab7A mediated mitophagy (Yan et al., 2021). Depleting different subunits of the ESCRT-III complex resulted in the inhibition of both Parkin dependent and independent mitophagy. Knockdown of CHarged Multivesicular body Protein 2A (CHMP2A) subunit of ESCRT-III complex resulted in the accumulation of unsealed mitophagosomes suggesting involvement of ESCRT complex protein in the closure of mitophagosome (Zhen et al., 2020) (Figure 2B).

### Mitophagosome—Lysosome Fusion

Unlike the canonical autophagy pathway, wherein several of the fusion machinery components such as SNAREs, Rabs and HOPS complex proteins are involved in the fusion of mature autophagosomes with the lysosomes (Nakamura and Yoshimori 2017), the fusion of mitophagosome with lysosome has been reported to require a different set of proteins. Parkin dependent mitophagy relies on Pleckstrin homology domain-containing family M member 1 (PLEKHM1) for the fusion of mitophagosomes with lysosomes (McEwan et al., 2015). PLEKHM1 has binding regions for several proteins involved in autophagic fusion such as Rab7, HOPS and Atg8 family of proteins (McEwan et al., 2015). PLEKHM1 is reported to preferentially interact with GABARAP to facilitate the fusion with lysosome in a PINK1-Parkin dependent manner (Nguyen et al., 2016) (Figure 2B). Although extensive research to elucidate pathways on receptor dependent and independent mitophagy is being done, mechanisms governing mitophagosome expansion and fusion especially in PINK1-Parkin independent mitophagy is underexplored and therefore requires detailed mechanistic dissection.

### Post-Translational Modifications in Mitophagy

#### Phosphorylation—Dephosphorylation Cascades

Phosphorylation and dephosphorylation of key mitochondrial and mitophagy-related proteins constitute one of the most important PTMs in the mitophagy pathway. The initial phosphorylation of Parkin by PINK1 at Ser65 in its ubiquitin domain triggers a feedforward loop recruiting more Parkin from the cytosol onto the mitochondrial surface (Ordureau et al., 2014; Harper et al., 2018) (as mentioned in the above sections). Another important mitophagy related kinase, TBK1, phosphorylates mitophagy adaptors, OPTN and NDP52 (Heo et al., 2015). Phosphorylation of OPTN at Ser177 facilitates its binding with Atg8 family of proteins, while phosphorylation at Ser residues 473 and 513 in its ubiquitin-binding region enhances its binding with Ub chains (Heo et al., 2015; Moore and Holzbaur 2016; Richter et al., 2016). TBK1 is also reported to phosphorylate Rab7A, thus aiding in the expansion of mitophagosome (Heo et al., 2018). ULK1, a key initiator kinase in autophagy process, localises to depolarised mitochondria. Interestingly, ectopic localisation of ULK1 complex on the mitochondrial surface was observed sufficient to induce mitophagy (Padman et al., 2019; Vargas et al., 2019). Leucine-rich repeat kinase 2 (LRRK2) and its constitutively active mutant (G2019S) are closely associated with Parkinson’s disease (PD). LRRK2 mediated phosphorylation of mitochondrial proteins positively regulates mitophagy. Onset of mitophagic cues triggers LRRK2 mediated phosphorylation of Miro proteins resulting in their removal from OMM, which arrests the trafficking of damaged mitochondrion (Hsieh et al., 2016). LRRK2 is also reported to phosphorylate Rab10 and OPTN leading to their recruitment on OMM, enhancing mitophagy (Wauters et al., 2020). Interestingly, overexpression of constitutively active LRRK2 disrupted the recruitment of Parkin and DRP1 onto OMM, thus inhibiting PINK1-Parkin mediated mitophagy (Bonello et al., 2019). Upon phosphorylation by IκB kinase α (IKKα), Autophagy and Beclin 1 Regulator 1 (AMBRA1) functions as a mitophagy receptor facilitating its interaction with Atg8 family of proteins, thereby, promoting mitophagy (Di Rita et al., 2018). Phosphorylation of Atg32 at Ser114 and Ser119 positions is mediated by CK2, and any impairment in its kinase activity is reported to suppress the interaction between Atg32 and Atg11, disrupting the mitophagy pathway (Kanki et al., 2013). Dephosphorylation mediated by different phosphatases counteract the effects of phosphorylation in mitophagy. Since, phosphorylation of Rab7A is an important event in mediating mitophagy, its dephosphorylation by PTEN (Phosphatase and Tensin homolog deleted on chromosome 10) might negatively regulate the process (Shinde and Maddika 2016). An isoform of PTEN, PTENL is involved in the dephosphorylation of Ub chains of Parkin, thereby reducing its translocation onto mitochondrial surface and suppressing mitophagy (Liang et al., 2015). Upon mitophagic induction, phosphoglycerate mutase family member 5 (PGAM5), dephosphorylates FUNDC1 which increases mitochondrial fragmentation through its interaction with DRP1 in a STX17 dependent manner (Sugo et al., 2018). Ppg1, a protein phosphatase 2A (PP2A)-like protein acting in concert with FAR complex proteins negatively regulate mitophagy by dephosphorylating Atg32, the mitophagy receptor in yeast cells (Furukawa et al., 2018).

#### Ubiquitination—Deubiquitination Cascades

Parkin has garnered a lot of attention and is one the most studied E3 ubiquitin ligases in the field of mitophagy. Parkin forms multiple Ub linkages such as poly-K6, -K11, -K48, and -K63 on its substrates including itself. K6 and K63 Ub linkages hold importance in mitophagy pathway in comparison to others as evidenced through Ub-replacement system based experimental studies (Ordureau et al., 2015). Mitochondrial ubiquitin ligase activator of NFKB 1 (MUL1), a E3 ubiquitin ligase is known to positively regulate mitophagy through ubiquitination of MFN1 and MFN2 (Yun et al., 2014). The membrane-associated RING-CH-type finger (MARCH5), another ubiquitin ligase, appears to have contrasting roles in mitophagy. Initial recruitment and activation of Parkin onto damaged mitochondria requires ubiquitination by MARCH5 (Koyano et al., 2019b), while on the other hand, it fine-tunes hypoxia induced mitophagy by ubiquitinating FUNDC1 receptor on OMM. This enhances its proteolytic degradation preventing exacerbated effects of mitophagy during hypoxia (Chen et al., 2017). Interestingly, ubiquitination of MARCH5 at lysine residue 268 by Parkin upon mitochondrial damage resulted in the extraction and subsequent translocation of MARCH5 from the damaged mitochondrion to peroxisomes. The extraction and translocation of MARCH5 is mediated by p97 and peroxisomal biogenesis factors 3 and 16 (Pex3 and Pex16). MARCH5 is a mitochondrion associated ubiquitin ligase, and this process thus aids MARCH5 to escape the amplified degradation of OMM proteins following mitochondrial damage (Koyano et al., 2019a). F-box only protein 7 (FBXO7) is reported to target TOM20, an outer mitochondrial membrane protein, promoting mitophagy (Teixeira et al., 2016) and has been reported to positively upregulate the mitophagy pathway by interacting with Parkin (Burchell et al., 2013). HECT, UBA and WWE domain containing E3 ubiquitin protein ligase 1 (HUWE1), like Parkin can ubiquitinate multiple Ub linkages and activate Parkin independent mitophagy pathway by inducing IKKα mediated phosphorylation at Ser1014 that subsequently activates AMBRA1 to interact with Atg8 family of proteins (Di Rita et al., 2018). Deubiquitination of Ub chains is achieved by the action of DUBs. USP30, USP8, USP13 and USP15 are some of the DUBs that have been studied for their role in mitophagy. Since DUBs antagonise the action of E3 ligases, it generally negatively regulates the mitophagy pathway except for USP8. USP30 has been extensively characterised as a DUB antagonising the action of Parkin, by deubiquitinating Parkin substrates such as Miro and TOMM20, thus, suppressing mitophagy (Bingol et al., 2014; Liang et al., 2015). USP313 directly interacts with Parkin and deubiquitinates it, while USP15, another DUB is reported to remove poly-K48 and -K63 Ub linkages from Parkin substrates without affecting the ubiquitination status of Parkin. Downregulation of USP13 or USP15 in mouse and *Drosophila* models of PD respectively, led to alleviation of PD-like phenotypes in these organisms (Cornelissen et al., 2014; Liu et al., 2019). USP8 unlike the other DUBs positively regulates mitophagy pathway by selectively removing K6-linked ubiquitin conjugates from Parkin, a process that enhances Parkin recruitment to mitochondrial surface, inducing mitophagy but on the contrary, a recent study reported rescue of PD like phenotypes in PINK1 knockout (KO) of flies, which requires further elucidation (von Stockum et al., 2019).

#### Other PTMs

Members of Sirtuin family of histone deacetylase have been reported to participate and upregulate mitophagy pathway. In an attempt to dissect out an interaction network of mitochondrial sirtuins, Yang *et al.,* employed a proteomics approach and identified SIRT3 to play a role in mitochondrial homeostasis. SIRT3 binds to ATP synthase in healthy mitochondria, but on mitochondrial membrane depolarisation, SIRT3 disassociates from ATP synthase, deacetylating mitochondrial matrix proteins (Yang et al., 2016). SIRT3 is also reported to directly deacetylate PINK1 and Parkin resulting in their induction, thus, promoting mitophagy (Huang et al., 2019). SIRT1 is involved in mitophagy through its NAD^+^–SIRT1–PGC-1α axis (Fang et al., 2014), and has also been reported to induce mitophagy by deacetylating PINK1 and Parkin (Yao et al., 2018). On the contrary, another study reported that the deficiency of SIRT1 upregulated PINK1-Parkin recruitment by supressing the activity of superoxide dismutase 2 (SOD2) (Di Sante et al., 2015). Other PTMs such as SUMOylation, glycosylation and acetylation have been sparsely reported in the context of mitophagy and has been discussed in an excellent review by Wang et al., 2020.

Given the mechanistic aspects of the proteins regulating the mitophagy pathway, extensive research performed in exploring the pathogenesis of mutations in mitochondrial proteins have laid ground for elucidating the involvement of mitophagy in them. The next part of the review aims to understand the imbalance in mitophagy pathway implicated in disease scenario, particularly focusing on neurodegenerative disorders.

## Unravelling the Molecular Mechanisms of Mitophagy Dysregulation in Neurodegenerative Diseases

Neurodegenerative diseases are a spectrum of complex heterogeneous disorders characterized by progressive degeneration of neurons and glia, thereby affecting the function of both central and peripheral nervous systems. Extensive research conducted in this field has helped delineate diverse molecular aspects, both of genetic and sporadic nature. In neurodegenerative diseases at organellar level, mitochondrial dysfunction has emerged as one of the predominant phenotypes. Whether damaged mitochondria are a cause or an effect of neurodegeneration is currently a hotly debated topic. In addition, reports on the accumulation of damaged mitochondria in most neurodegenerative diseases point towards the dysregulation of the mitophagy pathway. Here, we briefly try to address the known mechanistic details on mitophagy dysregulation and the open questions that need to be addressed to understand how this pathway is affected in neurodegenerative diseases.

### Alzheimer’s Disease

Alzheimer’s disease (AD), a progressive neurodegenerative disease with characteristic extracellular amyloid-beta plaques and intracellular Tau containing neurofibrillary tangles, is the leading cause of dementia worldwide. The clinical etiology of AD has been linked to both genetic and sporadic factors, leading to progressive degeneration of neurons, loss of memory, and cognitive decline of the affected individual (Long and Holtzman 2019). Mitochondrial dysfunction is one of the hallmarks of AD. Both amyloid-beta and pathogenic forms of Tau were found to interact with mitochondria, initiating a myriad of mitochondrial abnormalities ranging from impairment in electron transport chain (ETC), mitochondrial dynamics, ROS production, mitochondrial transport, lipid peroxidation, reduced ATP levels, and loss of Δψm (Chakravorty et al., 2019). Together with mitochondrial dysfunction, reports also suggest dysregulation of mitophagy pathway in AD.

#### Dysregulation of Mitophagy in AD

Dysregulation of mitophagy in AD. Impairment in the mitophagy pathway is a phenotype that has been closely associated with AD. A study by Fang et al. showed that levels of mitophagy related proteins such as Bcl2L13, PINK1, BNIP3L/NIX, p-ULK1 (Ser555), p-TBK1, FUNDC1, AMBRA1, and MUL1 is downregulated in AD patient brains and iPSC-derived cortical neuronal cultures generated from AD patients, indicative of defective mitophagy pathway (Fang et al., 2019). However, Parkin-mediated mitophagy was induced in the early stages of AD in mutant amyloid precursor protein transgenic (hAPP Tg) mouse models, where increased recruitment of Parkin and autophagic markers such as p62 and LC3 have been observed in the disease model compared to the wild type (Ye et al., 2015). Despite the initiation of mitophagy pathway in mutant hAPP Tg neurons, aberrant accumulation of mitophagosomes was also observed in these neurons, indicating the defective degradation of damaged mitochondria. Even though mitophagosomes marked with both p62 and LC3 showed a significant increase in association with LAMP1 positive vesicles, an increased retention of damaged mitochondria was observed in clustered and enlarged LAMP1 positive vesicles indicating that the degradative capacity of lysosomes in these mutant neurons is compromised (Ye et al., 2015). Studies demonstrated that mutations linked to familial AD (fAD) in presenilin 1 contributed to reduced lysosomal acidification due to defective maturation and transport of V-ATPase to the lysosomes, thereby compromising the degradative capacity of lysosomes (Ju-Hyun Lee et al., 2010). Additionally, presenilin 2 mutations associated with familial AD was shown to cause defective autophagosome-lysosome fusion by affecting Rab7 recruitment, which may contribute to the accumulation of auto/mitophagosomes in AD patients (Fedeli et al., 2019). Furthermore, a progressive reduction in Parkin expression was observed in both AD patient brains as well as mutant hAPP Tg mouse models, suggesting an impairment in effective activation of Parkin mediated mitophagy as the disease progressed (Ye et al., 2015).

Studies from Amadoro and Calissano’s group reported that expression of NH 2 -truncated hTau *in vitro* and *in vivo* AD models caused dysregulation in the mitophagy pathway by enhancing the recruitment of Parkin and Ubiquitin-C-terminal hydrolase L1 (UCHL-1) to the mitochondria. The authors also described that partially suppressing mitophagy in these models rescued the truncation induced neurotoxicity and restored mitochondrial density in the synapses (Corsetti et al., 2015). Contradictory to the observation of enhanced mitophagy in Tau AD models, recent investigations revealed that overexpression of human and pathogenic forms of Tau impaired mitophagy. For instance, Hu et al. showed that overexpression of hTau in both *in vitro* and *in vivo* model systems caused hyperpolarization of mitochondrial membrane potential and decreased recruitment of PINK1 and Parkin, leading to impaired mitophagy (Hu et al., 2016). Cummins et al. reported that overexpression of both hTau and frontotemporal dementia (FTD) mutant Tau (hP301L) blocked mitophagy in neuroblastoma cells by reducing Parkin translocation. A similar effect was observed in *C. elegans*, where the hTau overexpression reduced mitophagy levels, and mutant Tau inhibited mitophagy. According to this study, the underlying mechanism of reduced Parkin recruitment was not attributed to change in mitochondrial membrane potential but due to the aberrant interaction of Parkin with the projection domain of Tau, thereby sequestering Parkin in the cytosol (Cummins et al., 2019).

Apart from neurons, mitophagy in glial cells is also affected in AD. Among the glia, microglial cells from AD mouse hippocampus exhibited accumulation of damaged mitochondria with almost 60% reduction in mitophagy. As microglia perform high energy-dependent functions such as phagocytosis, accumulation of damaged mitochondria together with compromised mitophagy could impair microglial function, further contributing to AD pathogenesis (Fang et al., 2019). Collectively, studies indicate that the mitophagy pathway is affected at multiple steps, from recognition of damaged mitochondria, to their degradation in lysosomes. Additional investigations into the detailed mechanism of mitophagy impairment in specific AD models are required to validate and dissect the exact molecular mechanism of the defects in the pathway and its contribution to the establishment and progression of AD.

#### The Unknowns

Even though studies probing the mechanism of mitophagy impairment in AD, with a predominant focus on Parkin mediated mitophagy, are emerging recently, detailed mechanistic studies exploring the status of different mitophagy pathways, both Parkin dependent and independent, in AD models is still lacking. The predominant observation in reports that examine mutant APP and Aβ mediated mitophagy dysfunction is that mitophagy-related protein levels are significantly decreased in AD models. However, these studies reveal neither the mechanism behind the downregulated expression of mitophagy-related proteins nor the quantitative assessment of how the mitophagy flux is regulated in these models. While few studies on Tau mediated mitophagy dysfunction have probed into the underlying mechanism, additional research is required to provide more insights into the mechanism of mitophagy impairment in both sporadic and familial AD.

### Parkinson’s Disease

Parkinson’s disease (PD) is one of the common neurodegenerative diseases characterized by progressive loss of dopaminergic neurons in the substantia nigra pars compacta (SNpc), leading to classic Parkinsonian motor symptoms such as tremor, bradykinesia, rigid muscles, involuntary movements, and postural instability (Balestrino and Schapira 2020). Lewy bodies with aggregated α-synuclein accumulation in SNpc and other affected brain regions are considered as the pathological hallmarks of PD (Kalia and Lang 2015). PD has been closely associated with mitochondrial dysfunction and impairment in mitochondrial quality control, as the mutations known to cause familial PD are mostly in proteins that maintain mitochondrial homeostasis. Initial research that linked mitochondrial dysfunction to PD was based on the observation that toxins such as 1-methyl-4-phenyl-1,2,3,6,-tetrahydropyridine (MPTP), that inhibit mitochondrial complex I function, caused loss of dopaminergic neurons leading to Parkinsonism. Moreover, PD-affected patient brains also manifested decreased mitochondrial complex I activity, thereby associating mitochondrial dysfunction with PD (Davis et al., 1979; Langston et al., 1983; Schapira et al., 1990). A recent report by Rodríguez et al. described that disrupting the mitochondrial complex I in mouse dopaminergic neurons triggered a progressive loss of the dopaminergic phenotype accompanied by deficits in motor learning, reaffirming that mitochondrial complex I dysfunction is sufficient to cause human-like Parkinsonism in mouse models (González-Rodríguez et al., 2021). Mitochondrial dysfunction in PD has also been triggered by pathogenic α-synuclein that preferentially interact with mitochondria bringing about a block in mitochondrial protein import, mitochondrial membrane depolarisation, mitochondrial permeability transition pore (MPTP) opening, impairment in ETC, increased ROS, and induction of mitochondrial fragmentation (Malpartida et al., 2021). Finally, along with mitochondrial dysfunction, mitophagy pathway is also affected in PD.

#### Dysregulation of Mitophagy in PD

Impairment of mitophagy was initially associated with PD based on the observation that PD patient brain samples showed accumulation of autophagosomes containing damaged mitochondria (Zhu et al., 2003). In addition, mutations in PINK1 and Parkin that are the predominant proteins involved in mitophagy, were shown to contribute to the early onset of autosomal recessive PD (Valente et al., 2004; Lücking et al., 2000). Examining the mechanisms of both PINK1 and Parkin mutations showed that mutant proteins impair mitophagy by affecting different stages of the pathway. The mutation R42P in Parkin blocked its recruitment to damaged mitochondria upon stress, whereas R275W Parkin mutant localised to depolarised mitochondria but did not induce perinuclear mitochondrial aggregation. On the contrary, mutations A240R and T415N in Parkin neither blocked its recruitment to depolarized mitochondria nor the formation of mitochondrial aggregates, but instead decreased the ubiquitination of damaged mitochondria. This further impaired the recruitment of p62 and HDAC6 thereby preventing the clearance of damaged mitochondria (Joo-Yong Lee et al., 2010). Similar results of decreased ubiquitination and clearance of OMM proteins upon stress was observed in patient derived cells expressing PINK1 and Parkin homozygous mutations (Rakovic et al., 2011). Recently, evidences from PD patient brains showed enhanced pS65-Ub levels in sporadic PD. However, substantia nigra from Parkin and PINK1 mutant cases showed diminished pS65-Ub levels reiterating the impairment of mitophagy pathway in PD (Hou et al., 2018). The loss of function of PINK and Parkin leading to PD-like phenotype was recapitulated in *Drosophila* and rat models. In PINK1 or Parkin loss of function model in *Drosophila*, mitophagy pathway was impaired leading to accumulation of swollen and damaged mitochondria with disintegrated cristae, dopaminergic degeneration, locomotion defects, severe flight muscle degeneration, male sterility, and reduction in life span (Greene et al., 2003; Clark et al., 2006; Whitworth et al., 2005; Cornelissen et al., 2018). Similarly, PINK1 KO rat models exhibited dopaminergic cell loss, progressive nigral neurodegeneration and motor deficits further pointing toward the role of impaired mitophagy in developing PD pathology. Surprisingly, Parkin loss of function models in rat displayed normal behaviour without any pathological phenotype indicating the possible involvement of other ubiquitin ligases that compensate the loss of Parkin (Dave et al., 2014). Contrary to the observations in *Drosophila* and rat models, PINK1 and Parkin KO mouse models exhibited very subtle or no robust PD related phenotypes (Perez and Palmiter 2005; Kitada et al., 2007). The possible explanation could be the presence of compensatory mitochondrial quality control mechanisms or activation of PINK1-Parkin independent mechanisms that could alleviate the expected phenotype, in a species-specific manner. However, when these KO mice were challenged with stressors, the results similar to the *Drosophila* and rat models were obtained. The stressors were either genetically created by crossing with mtDNA mutator mice and thereby inducing mitochondrial DNA mutations or by inducing intestinal bacterial infection (Pickrell et al., 2015; Matheoud et al., 2019).

Apart from mutations in PINK1 and Parkin, mutations in several other genes associated with PD pathogenesis also show impairment in mitophagy pathway. Mutations in the leucine-rich repeat kinase 2 (LRRK2, PARK8) gene is known to cause autosomal dominant form of PD and accounts for most of familial PD cases (Tolosa et al., 2020). The pathogenic mutations of LRRK2 were observed to have hyperphosphorylated kinase activity and emerging reports suggest that these mutations impair the mitophagy pathway thereby contributing to PD pathogenesis (Singh and Ganley 2021). During PINK1-Parkin mitophagy, the transport of damaged mitochondria is arrested by degradation of Miro1, a component of the primary motor/adaptor complex that helps in anchoring the motor protein kinesin to the mitochondria, in a PINK1-Parkin dependent manner (Wang et al., 2011). LRRK2 aids this process by forming a complex with Miro1 and targeting it for degradation *via* the proteasome. This function of LRRK2 was shown to be disrupted in PD patient-iPSC-derived neurons with pathogenic LRRK2G2019S mutation. In patient cell lines, the mutant LRRK2 does not interact with Miro1, thereby resulting in reduced degradation rates of Miro1 which slows down the arrest of damaged mitochondria and further delays the initiation of mitophagy (Hsieh et al., 2016). Two independent investigations conducted in patient-derived cells with LRRK2 mutations showed reduced mitophagy flux (Bonello et al., 2019; Korecka et al., 2019). Additional research revealed new insights into the mechanism of mitophagy impairment in LRRK2 mutations. While Bonello *et. al.* uncovered that mutant LRRK2-G2019S disrupts interactions between Parkin and OMM proteins in a kinase dependent manner, Wauters *et. al.* identified a new mechanism wherein the two LRRK2 mutations (G2019S and R1441C), impaired mitophagy by decreasing the recruitment of OPTN to damaged mitochondria (Bonello et al., 2019; Wauters et al., 2020). This was mediated through enhanced phosphorylation of threonine 73 of Rab 10 by the mutant LRRK2 which disrupted the interaction of Rab10 with OPTN thereby restricting its recruitment to damaged mitochondria (Wauters et al., 2020). The impairment in mitophagy flux due to LRRK2 mutations was recapitulated in mouse models carrying the G2019S mutation, where reduced mitophagy flux was observed in dopaminergic neurons and microglia in brain, validating the effect of LRRK2 mutations in mitophagy pathway (Singh et al., 2021).

A recent study by Grossmann *et. al.* identified mutations in Miro1 that are linked to PD pathogenesis (Grossmann et al., 2019). In addition to its role in mitophagy, Miro1 is also known to be involved in modulating mitochondrial calcium homeostasis and in maintaining the integrity of ER-mitochondria contact sites (Lee et al., 2016). Miro1-R272Q mutant iPSC-derived neurons exhibited decreased mitochondrial motility, reduced capacity to modulate calcium levels in response to increase in cytosolic calcium, alteration in ER-mitochondria contact sites and impaired mitophagy flux (Berenguer-Escuder et al., 2019; Berenguer-Escuder et al., 2020). Further studies are required to understand the mechanistic aspects of the mitophagy block in Miro1 PD associated mutants and questions pertaining to this are yet to be addressed.

As lysosomes are the end points for the degradation of damaged mitochondria *via* mitophagy, PD associated mutations that affect the function of lysosomes also impair mitophagy, further contributing to the PD pathogenesis. Heterozygous mutations in glucosylceramidase beta (GBA), a lysosomal enzyme that converts glucosylceramide to ceramide and glucose, is one of the most common genetic risk factors for PD pathogenesis, contributing to 7–20% of all PD cases (Stoker et al., 2018). Interestingly, homozygous mutations in GBA cause Gaucher disease (GD), the most common lysosomal storage disorder. Studies conducted in conditional KO mouse models of GBA reported decreased mitochondrial membrane potential, mitochondrial fragmentation, reduced complex I, II and III activities, decrease in oxygen consumption rate and impaired mitophagy in neurons and astrocytes, in addition to the compromised lysosomal activity (Osellame et al., 2013). A recent study by Li *et. al.* investigated the effect of PD associated heterozygous mutation of GBA L444P, in a knock-in mouse carrying one copy of the L444P mutant *Gba* allele (*Gba*L444P/WT) (Hongyu Li et al., 2019). Neurons expressing L444P mutation exhibited decreased mitochondrial membrane potential, increased ROS levels and mitochondrial mass suggesting mitochondrial dysfunction and defective clearance of damaged mitochondria. The study also explored the status of mitophagy and found that mutant neurons expressing L444P mutation were impaired in the recruitment of Parkin, NBR1, and LC3 to damaged mitochondria and had reduced mitophagy flux reiterating the impairment of mitophagy pathway (Hongyu Li et al., 2019). Initiation of autophagy and lysosomal function was also impaired in the mutant neurons. The mitochondrial pathology was further analysed in the brains of PD patients with L444P mutation, wherein increased level of mitochondrial proteins, defects in respiratory complex functions and impaired autophagy was observed, further corroborating the results obtained in the mouse models (Hongyu Li et al., 2019).

Collectively, mitochondrial dysfunction and impairment in the mitophagy pathway can be considered as one of the strong candidates contributing to PD pathogenesis, owing to its predominant occurrence in various PD models. Therefore, therapeutic interventions targeting mitochondrial health and mitophagy together with the existing PD medications could be developed as potential treatment strategies for alleviating PD pathogenesis.

#### The Unknowns

Since the discovery of the link between PD and the key players of mitophagy pathway - PINK1 and Parkin had been made, extensive research has gone into elucidating the pathway and identifying the effect of PD associated mutations on PINK1-Parkin pathway. Mechanistic details of mitophagy impairment in different PD models carrying specific mutations are emerging recently. While mutations in key genes like PINK1, Parkin, LRRK2, GBA are explored in more detail, there still remains the need for investigations in greater depth to identify the mechanisms in less explored PD associated genes. As PINK1 and Parkin KO mice fail to exhibit PD related phenotypes, the existence of compensatory mitophagy pathways that are PINK1-Parkin independent requires further exploration. Additionally, detailed research is also required in exploring the status of mitophagy pathway in sporadic PD. Understanding the molecular mechanisms of mitophagy impairment in these models will help to devise efficient therapeutic strategies to alleviate the disease.

### Huntington’s Disease

Huntington’s disease (HD) is an autosomal dominant neurodegenerative disorder caused by the abnormal expansion of CAG repeats that encodes the polyQ tract at the N- terminus of the huntingtin protein. In the normal population, CAG repeats of huntingtin protein ranges from 6 to 35 units, whereas the affected individuals show CAG repeats more than 35 units which progressively triggers the disease process leading to characteristic disease symptoms such as chorea, dystonia, impaired gait and posture, cognitive decline and psychiatric disorders (Bates et al., 2015). Mutant huntingtin (mHTT) protein can cause dysfunction of multiple intracellular processes such as proteostasis, transcription and cell signaling, endocytic and secretory pathways, intracellular trafficking, and mitochondrial homeostasis (Wanker et al., 2019). Similar to other neurodegenerative disorders mitochondrial health and quality control is reported to be dysregulated in HD. Mitochondrial dysfunction in HD causes decreased mitochondrial biogenesis due to downregulation of PGC-1α, altered functioning of respiratory chain complexes II, III, and IV, increased ROS production, enhanced mitochondrial fragmentation due to interaction of mutant HTT with DRP1, impaired mitochondrial trafficking, and inhibition of mitochondrial protein import due to interaction of mHTT with TIM23 complex, thereby leading to disruption of mitochondrial homeostasis (Johri et al., 2013; Yablonska et al., 2019). Reports also suggest dysregulation of mitophagy in HD, the details of which are discussed below.

#### Dysregulation of Mitophagy in HD

A study by Khalil *et.al.* described the accumulation of damaged mitochondria in *Drosophila* expressing mHTT (Httex1p Q93), suggesting an impairment in its clearance (Khalil et al., 2015). Similar results were observed in mouse striatal HdhQ7 and HdhQ111 cell lines with CAG repeats 7 and 111 respectively, wherein degradation of damaged mitochondria was impaired. Further research into the mechanism uncovered that Parkin recruitment and subsequent ubiquitination was unaffected, but the colocalization of ubiquitinated mitochondria with LC3 was significantly decreased in HdhQ111 versus HdhQ7 cells, suggesting an impairment in the targeting of damaged mitochondria to autophagosomes (Khalil et al., 2015).

mt-Keima mouse model expressing human Huntingtin’s transgene (HTT) also showed marked reduction in mitophagy (Sun et al., 2015). A study by Martinez-Vicente *et. al.* reported that interaction of mHTT with p62, an autophagy adaptor, disrupted the transport of autophagosomes in neurons, possibly contributing to ineffective clearance of damaged mitochondria (Martinez-Vicente et al., 2010). In addition, mHTT interaction with GAPDH disrupted trafficking of the damaged mitochondria resulting in dysfunctional micro-mitophagy (Hwang, Disatnik, and Mochly-Rosen 2015). A recent study investigating the mechanism of mitophagy impairment in HD described that in differentiated striatal ST-Q111 cells (111 CAG repeats), mHTT impaired the initiation of mitophagy by increasing the stability of ULK1 and mTOR complex thereby maintaining ULK1 in its inactive form (Franco-Iborra et al., 2021). mHTT further interacts with Beclin1 (BECN1) leading to its degradation and hence downregulates the formation of the BECN1-PIK3R4/VPS15 complex. Further findings from the study reported that mHTT did not affect the ubiquitination of damaged mitochondria but impaired the interaction of mitophagy adaptors OPTN, CALCOCO2, SQSTM1/p62 and NBR1 with LC3 (Franco-Iborra et al., 2021). Contrary to the studies showing mitophagy impairment in HD, an independent study from Guo *et. al.* reported that mHTT recruits valosin-containing protein (VCP) to the mitochondria, which promotes excessive mitophagy leading to neuronal death (Guo et al., 2016). However, further studies investigating the effect mHTT on mitophagy by different research groups showed an impairment in the pathway. Therefore, detailed research investigating the mechanisms of mitophagy dysregulation in HD is required and findings from these studies will provide more insights on proteins involved in the mitophagy impairment in HD, which can then be used for developing potential therapeutic strategies.

#### The Unknowns

In the recent years, studies probing the mechanism of mitophagy impairment in multiple HD model systems have been emerging. Independent studies showed that mHTT affected multiple steps in the mitophagy pathway such as initiation, cargo recognition, interaction with autophagosomes and transport of autophagosomes, thereby impeding the clearance of damaged mitochondria. The major studies have predominantly focused on PINK1-Parkin mitophagy although the status of Parkin independent mitophagy pathways in HD remains unexplored. Also, comprehensive mechanistic studies probing the effect of varying number of CAG repeats on mitophagy impairment is lacking. These detailed studies, if performed, can provide insights into the degree of mitophagy impairment caused due to the varying number of CAG repeats - an information which can then be used to develop effective and personalized therapeutic strategies to ameliorate the HD pathology.

### Amyotrophic Lateral Sclerosis

Amyotrophic lateral sclerosis (ALS) is a neurodegenerative disease characterized by progressive degeneration of motor neurons that arise from both brain and spinal cord, thereby causing muscle weakness, muscular atrophy, dysarthria and paralysis in affected individuals (Taylor et al., 2016). Approximately 10% of the ALS cases are familial and mostly follow an autosomal dominant inheritance pattern, while the rest are sporadic. Mutations of over 20 genes have been associated with ALS - the prominent ones being C9orf72, superoxide dismutase 1 (SOD1), fused in sarcoma (FUS), TAR DNA binding protein (TDP-43), OPTN, TBK1 and SQSTM1/p62, that accounts for 60% of familial and 11% of sporadic ALS cases. As the genes associated with ALS are involved in multiple cellular processes, the possible pathogenic mechanisms of ALS include dysregulation of proteostasis, RNA toxicity, defective axonal transport, oxidative stress, excitotoxicity, and mitochondrial dysfunction, thereby making ALS a multifactorial disease (Taylor et al., 2016). In ALS patient motor neurons, accumulation of aggregated, swollen and vacuolated mitochondria have been observed (Ruffoli et al., 2015). Further investigations showed a multitude of ALS associated mitochondrial defects such as defective oxidative phosphorylation, increased ROS production, impaired mitochondrial dynamics and transport, disrupted ER-mitochondria contact sites, dysregulated mtDNA transcription and defects in calcium buffering capacity (Smith et al., 2019). In addition to mitochondrial defects, mitophagy pathway is also reported to be compromised in ALS, which further contributes to the pathogenesis.

#### Dysregulation of Mitophagy in ALS

Impairment in mitophagy pathway is emerging as a predominant phenotype contributing to the pathophysiology of ALS (Madruga et al., 2021). In ALS mouse models expressing SOD1^G93A^ mutation, while degenerated mitochondria accumulated in neuromuscular junctions (NMJs), a concomitant increase in the number of mitophagosomes was not observed, suggesting compromised mitophagy. Furthermore, levels of mitophagy proteins like Parkin, PINK1, BNIP3 and p62 was also reduced in SOD1^G93A^ mice (Rogers et al., 2017). A recent examination of the mechanism of mitophagy impairment in SOD1 mutations showed that N2A cells expressing A4V and G93A mutations exhibited strong interactions with the mitophagy adaptor OPTN. Moreover, it was observed that OPTN is sequestered in SOD1 mutant aggregates and this leads to a decrease in mitophagy flux in cells expressing the mutants (Tak et al., 2020). On the contrary, a study by Palomo *et. al.* reported enhanced mitophagy flux in the spinal cords of SOD1^G93A^ mouse model. Surprisingly the mutant mice manifested reduced levels of Parkin and KO of Parkin in these mice reduced the protein aggregates, delayed motor neuron loss, decreased NMJ degeneration and increased life span (Palomo et al., 2018). The authors propose that in ALS, Parkin mediated mitophagy may be neuroprotective in the initial stages of disease progression. However, elevated mitophagy flux if sustained for a prolonged duration could have a negative effect on the neuronal survival due to enhanced depletion of mitochondria. Therefore, detailed studies are required to understand the exact status of mitophagy in the SOD1 mutant models. Impaired mitophagy in ALS can also be due to mutations in adaptor proteins. For example, about 40 fALS associated mutations have been observed in OPTN, an autophagy/mitophagy specific adaptor (Ayaki et al., 2018; Madruga et al., 2021). In cells expressing OPTN mutant E478G, the recruitment of the mutant OPTN to damaged mitochondria was disrupted, thereby impairing the clearance of damaged mitochondria (Wong and Holzbaur 2014). Further studies in motor neuron-like NSC-34 cells showed that the mutations Q398X and E478G in OPTN impeded its ability to interact with myosin VI leading to its abnormal diffused cytoplasmic expression. In these mutants, mitophagosome-lysosome fusion was also blocked, resulting in the accumulation of mitophagosomes (Sundaramoorthy et al., 2017). Mutations in another autophagy adaptor, SQSTM1/p62 are also linked to ALS pathogenesis (Fecto et al., 2011). Investigations in patient fibroblasts carrying ALS linked p62 mutations and SH-SY5Y cells with loss of function of p62 exhibited a decrease in mitochondrial membrane potential, increased ROS production, reduced complex I activity indicating compromised mitochondrial health (Bartolome et al., 2017). However, the mechanism of the effect of ALS linked SQSTM1/p62 mutations on mitophagy pathway is yet to be explored. For efficient binding of OPTN and p62 to ubiquitinated cargo, the adaptors undergo a post translational modification by a serine-threonine kinase, TBK1. About 80 mutations are reported in the TBK1 gene that are associated with ALS pathogenesis (Oakes et al., 2017; Madruga, Maestro, and Martínez 2021). Mutations in TBK1 affect mitophagy pathway at different stages, for example, mutations E696K, M559R, G217R in TBK1 impaired its recruitment to damaged mitochondria, p.690-713del and E696K in the CCD2 domain of TBK1, inhibited its interaction with OPTN. Additionally, G217R and M559R-TBK1 mutants blocked the recruitment and formation of LC3 rings on damaged mitochondria (Harding et al., 2021; Freischmidt et al., 2015). Even though studies exploring the mechanisms of mitophagy impairment in ALS are emerging, detailed research is required to clear the contradictions and to understand the contribution of mitophagy impairment in the pathophysiology of ALS.

#### The Unknowns

Investigations into the mechanisms of mitophagy dysregulation in ALS, suggests that multiple stages such as cargo recognition, adaptor recruitment and interaction of damaged mitochondria with mitophagosomes are impaired. Currently, the effect of mutations in only a few genes such as OPTN, TBK1 and SOD1 are being explored. Further studies on other ALS associated genes linked to mitophagy impairment are essential to achieve a detailed understanding on the effect of specific mutations on the pathway. As with the other neurodegenerative diseases, status of parkin independent mitophagy in ALS still remains to be explored. Contrary to impaired mitophagy, an independent study using SOD1 mouse model described that prolonged enhancement of mitophagy due to persistent mitochondrial damage could be detrimental to neuronal survival. If modulation of mitophagy as a therapeutic strategy for ALS has to be developed, detailed investigations are required to explore both the dysregulation of mitophagy in different stages of disease progression and to understand the contradictory reports obtained in the disease models.

**FIGURE 3:**
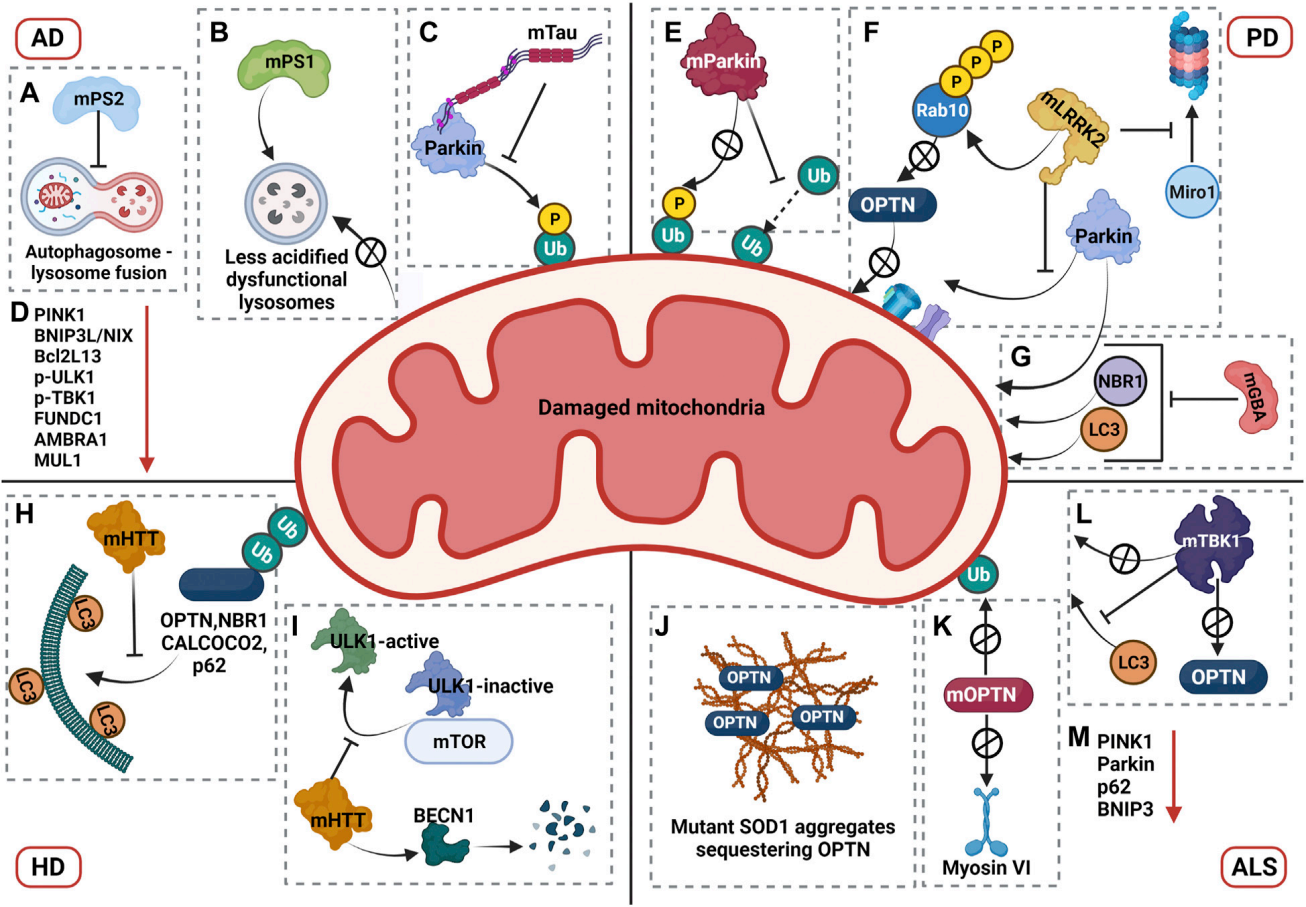
Mechanisms of mitophagy dysregulation in neurodegenerative disorders. **AD**: **(A)** AD associated mutations in presenilin 2 (mPS2) blocks mitophagosome to lysosome fusion by affecting the Rab7 recruitment, leading to accumulation of mitophagosomes. **(B)** AD associated mutations in presenilin 1 (mPS1) gene decrease the lysosomal acidification by impairing the transport of V-ATPase thereby blocking the clearance of damaged mitochondria. **(C)** Pathogenic Tau (mTau) interacts with Parkin with its projection domain, inhibiting the recruitment of Parkin to damaged mitochondria. **(D)** AD models also show down regulation of mitophagy associated proteins PINK1, BNIP3L/NIX, Bcl2L13, p-ULK1, p-TBK1, FUNDC1, AMBRA1, and MUL1 (The red arrow in the figure represents downregulation). **PD**: **(E)** PD associated mutations affect the role of Parkin in two ways. Mutations either block Parkin recruitment to damaged mitochondria or impair the ubiquitination capacity of Parkin, both of which impairs the mitophagy pathway. **(F)** PD associated mutations in LRRK2 impair mitophagy pathway at different stages. Based on specific mutations, LRRK2 hyperphosphorylates Rab10, which prevents its interaction with OPTN thereby blocking its recruitment to damaged mitochondria. Mutant LRRK2 has been shown to prevent the interaction of Parkin with OMM proteins. Mutant LRRK2 also inhibits Miro1 degradation, an essential step for arrest of damaged mitochondria, further impairing the pathway. **(G)** PD associated mutations in GBA inhibit recruitment of Parkin, NBR1 and LC3 to the damaged mitochondria. **HD**: Dysregulation of mitophagy pathway by mutant HTT (mHTT) involves multiple mechanisms. **(H)** mHTT blocks the recruitment of OPTN, NBR1, CALCOCO2 and p62. **(I)** mHTT stabilizes the interaction of inactive ULK1 to mTOR, thereby impairing the formation of autophagy initiation complex. mHTT promotes degradation of Beclin1, which also prevents the formation of autophagy initiation complex. **ALS**: **(J)** Mutant SOD1 (mSOD1) aggregates sequester OPTN preventing its recruitment to damaged mitochondria. **(K)** ALS associated mutations in OPTN (mOPTN) blocks its recruitment to damaged mitochondria and also impairs its interaction with myosin VI, thereby restricting the localization of mutant OPTN in cytoplasm. **(L)** ALS linked mutations in TBK1 (mTBK1) blocks its recruitment to damaged mitochondria, impairs its interaction with OPTN and impedes the recruitment of LC3 to damaged mitochondria. **(M)** In addition, mitophagy related proteins PINK1, Parkin, p62, and BNIP3 are downregulated in ALS (The red arrow in the figure represents downregulation).

In conclusion, mitophagy dysregulation is emerging as one of the predominant contributing factors in the pathophysiology of neurodegenerative disorders. Studies elucidating the mechanism of impaired mitophagy provide new insights in understanding how this pathway is affected in specific disease conditions (Figure 3). Apart from the major neurodegenerative disorders discussed above, role of mitophagy is emerging in other neurodegenerative disorders such as multiple sclerosis (MS), vascular dementia (VD), mixed dementia (MD), and ataxia telangiectasia (A-T). Both MS and VD disease models showed elevated levels of autophagy and mitophagy related proteins in patient sera as well as enhanced mitophagy. While A-T disease models exhibited defective mitophagy, MD patients sera showed reduced levels of autophagy and mitophagy proteins (Fang et al., 2016; Castellazzi et al., 2019; Hassanpour et al., 2020; Patergnani et al., 2021; Zheng et al., 2021). However, mechanisms of mitophagy dysregulation in these models are yet to be explored. Information gleaned from studies investigating the mechanisms of mitophagy dysregulation using different mitophagy reporters and model systems (Table 1, Box 1) can be further used to devise potential therapeutic strategies to alleviate neurodegenerative disorders. In this direction, emerging literature focusing on pharmacological interventions in neurodegenerative disorders has been summarized in Table 2.

**TABLE 1:** Reporter proteins and model systems used to study mitophagy.

Sl. No	Reporter protein	Model system and cell lines	Reference
1	mito-QC	SH-SY5Y	Allen et al. (2013)
2	mt-mKeima	HeLa	Sun et al. (2017)
3	mito-QC	U2OS	Allen et al. (2013)
4	mito-SRAI	MEF, H4	Katayama et al. (2020)
5	mito-Rosella	*Caenorhabditis elegans*	Palikaras, Lionaki, and Tavernarakis (2015), Cummins et al. (2019)
6	mt-mKeima	*Drosophila melanogaster*	Lee et al. (2018)
7	mito-QC		Lee et al. (2018)
8	mt-mKeima	*Danio rerio*	Wrighton et al. (2021)
9	mito-EGFP mCherry		Wrighton et al. (2021)
10	mito-QC	*Mus musculus*	McWilliams et al. (2016)
11	mt-mKeima		Sun et al. (2015)
12	Mito-Timer		Wilson et al. (2019)

Box 1 **Fluorescent reporter proteins for monitoring mitophagy**

1) Tandemly tagged pH sensitive reporters to analyze mitophagy flux.
  a. **mito-QC:** A pH sensitive mitophagy reporter protein with a tandem mCherry-GFP tag is attached to mitochondrial targeting sequence (MTS) of FIS1 (residues 101–152), an OMM protein Allen et al. 2013.
  b. **mito-Rosella:** A mitophagy reporter protein with pH-insensitive RFP variant, DsRed.T3 and a pH-sensitive GFP variant, pHluorin, tandemly tagged to MTS of citrate synthase at its N-terminus Rosado et al., 2008.
  c. **mito-mRFP-EGFP:** A pH-sensitive reporter protein constructed by inserting MTS sequences of human cytochrome C oxidase subunit VIII, N-terminally in frame with mRFP-EGFP Kim et al., 2013.
  All the above-mentioned reporter proteins share similar principle for monitoring mitophagy flux. Upon induction of mitophagy, mitochondria expressing the tandem reporters are targeted to lysosomes, where the pH sensitive (GFP, pHluorin, EGFP) fluorophores get quenched and pH insensitive (mCherry, DsRed.T3, mRFP) fluorophores show stable expression. This differential quenching property of these tandem mitophagy reporters help to visualize and quantitate the mitochondria targeted to lysosomes as a read out of mitophagy flux.
2. **mt-mKeima:** A pH sensitive mitophagy reporter protein developed by tagging tandem repeats of COX VIII MTS to mKeima. Keima is pH sensitive protein exhibiting dual excitation spectra and is stable at lysosomal pH. Upon mitophagy induction, the mitochondria expressing mt-mKeima targeted to lysosomes show an excitation peak at 550 nm whereas the mitochondria in cytosol shows an excitation peak at 438 nm. The dual excitation of the reporter helps to distinguish the mitochondria in lysosomes and cytosol, and hence can be used as a readout for mitophagy Katayama et al., 2011.
3. **MitoTimer:** A fluorescent reporter protein constructed by tagging MTS of COX VIIIA to DsRed1-E5 (Timer). The fluorescence of DsRed1-E5 shifts from green to red, as the protein matures Terskikh et al., 2000. The MitoTimer, therefore can be used as reporter for spatio-temporal monitoring of mitochondrial turnover and dynamics Hernandez et al., 2013.
4. **mito-SRAI (Signal Retaining Autophagy Indicator):** A novel fluorescent mitophagy reporter protein constructed by fusing a tandem repeat of cytochrome C oxidase subunit VIII MTS to N-terminus of TOLLES-YPet (SRAI) fusion construct. TOLLES (TOLerance of Lysosomal EnvironmentS) is an acid-fast CFP that is stable at lysosomal pH whereas YPet is a YFP variant that is both acid and protease sensitive and hence gets degraded in lysosomes. Due to the differential stability of these fluorescent proteins in lysosomes, mito-SRAI can be used as a mitophagy reporter that can distinguish and quantitate the mitochondria in cytosol and lysosomes Katayama et al., 2020.

**TABLE 2:** Therapeutic interventions in neurodegenerative disorders (ND) *via* the modulation of mitophagy pathway.

ND	Compounds	Related pathway	Model used in the study	Reference
Alzheimer’s disease	Rapamycin	mTOR pathway	Apolipoprotein E4 transgenic mice, AAV-based mouse model of early-stage AD-type tauopathy, 3xTg-AD, Tg2576 mice, primary neuronal cell culture	Majumder et al. (2011), Tian et al. (2011), Caccamo et al. (2014), Siman, Cocca, and Dong (2015), Lin A. L. et al. (2017)
	Memantine	GSK3β mediated phosphorylation	Tg2576 mice and 3xTg-AD mice	Martinez-Coria et al. (2010)
	Metformin	AMPK pathway	P301S transgenic mouse	Barini et al. (2016)
	Resveratrol	Enhancement of mitophagy pathway	Aβ_1–42_ in PC12 cells	Wang et al. (2018)
	NMN (Nicotinamide mononucleotide)	Precursor of NAD^+^, enhances deacetylation of autophagy related proteins through SIRT1 pathway and regulates JNK pathway	APPswe/PS1dE9 (AD-Tg) mice	Fang et al. (2014), Yao et al. (2017)
	NR (Nicotinamide riboside)	Precursor of NAD^+^, decreases Aβ toxicity through PGC-1α-mediated degradation of BACE1 and activation of SIRT1, FOXO signalling and mitoUPR pathways	Tg2576 mice, *C. elegans* and HEK293T cell line	Cantó et al. (2012), Gong et al. (2013), Mouchiroud et al. (2013)
	NAM (Nicotinamide)	Increases mitochondrial integrity through SIRT1, CREB, Akt and MAPK activation	3xTgAD mice and primary rat cortical neuronal culture	Dong Liu et al. (2013)
	Urolithin A	Upregulation of mitophagy proteins such as PINK1-PRKN, AMBRA1, FUNDC1, OPTN and autophagy pathway proteins	APP/PS1 mouse model, SH-SY5Y cells and *C. elegans*	Fang et al. (2019), Ryu et al. (2016)
	Actinonin	Upregulation of PINK1 expression and enhanced mitophagy	SH-SY5Y cells, *C. elegans* and APP/PS1 transgenic mouse	Fang et al. (2019)
	Nilotinib	Elevates expression of Parkin and enhances its interaction with Beclin1 resulting in amyloid clearance	C57BL/6 mice injected with Aβ_1–42_	Lonskaya et al. (2014)
Parkinson’s disease	Metformin	AMPK pathway	SH-SY5Y cells and MPTP treated mice	Lu et al. (2016)
	Kinetin triphosphate	Upregulation of PINK1 activity	SH-SY5Y cells and rat primary neuronal culture	Hertz et al. (2013)
	SR3677	ROCK inhibitor, induces HK2 mediated recruitment of Parkin	*Drosophila melanogaster* model treated with PD-causing toxin paraquat and differentiated SH-SY5Y cells	Moskal et al. (2020)
	T0466 and T0467	Activation of PINK1-Parkin pathway	Differentiated dopaminergic neurons and myoblasts from human iPSCs, *Drosophila melanogaster*	Shiba-Fukushima et al. (2020)
	T-271	Parkin dependent induction of mitophagy pathway	H4 cells	Katayama et al. (2020)
	Rapamycin	mTOR pathway and inhibition of RTP801 induction	Mice treated with MPTP to induced Parkinsonism, PC12 cells	Malagelada et al. (2010), Kangyong Liu et al. (2013)
	PMI (p62 mediated mitophagy inducer)	Induces signalling and expression of p62	SH-SY5Y and MEF cells	East et al. (2014)
	Carnosic acid (CA)	Induction of PINK1-Parkin pathway	SH-SY5Y cells	Lin and Tsai (2019)
	Salidroside	Induction of PINK1-Parkin mitophagy pathway	C57BL/6 mice treated with MPTP	Li and Chen (2019)
Huntington’s disease	Rapamycin and CCI-779 (Rapamycin analog)	mTOR pathway	PC12 and COS7 cells, HD model of *Drosophila melanogaster* and mice	Ravikumar et al. (2004)
	Thiazolidinedione and rosiglitazone	Agonist of PPARγ, enhanced recruitment of PPARγ to Htt aggregates	R6/2 mice and N2A cell line	Chiang, Chern, and Huang (2012)
	Metformin	Activation of AMPK pathway	128Q HD model of *C. elegans,* HdhQ111 knock-in mouse model, and HD patients	Vázquez-Manrique et al. (2016), Hervás et al. (2017)
	Trehalose	Upregulation of autophagy pathway resulting in reduced ROS	R6/2 transgenic mice and fibroblasts derived from HD patients	Tanaka et al. (2004), Fernandez-Estevez et al. (2014)
	HV-3 peptide	Inhibition of mtHtt and VCP interaction, thereby preventing VCP induced excessive mitophagy	HdhQ111 striatal cells, HD patient derived fibroblasts and R6/2 mice model	Guo et al. (2016)
Amyotrophic lateral sclerosis	Trehalose	Upregulation of autophagy genes such as *Lc3, Atg5, Beclin1,* and *p62*	SOD1^G86R^ and SOD1^G93A^ transgenic mice model of ALS	Castillo et al. (2013), Zhang et al. (2014)
	Rilmenidine	Elevated levels of mitophagy	NSC-34 cell culture and SOD1^G93A^ transgenic mouse model of ALS	Perera et al. (2018)

## Conclusion

Mitophagy pathway encompasses autophagy-mediated degradation of damaged mitochondria. Scientific contributions spanning over 2 decades have unraveled several molecular players governing mitophagy, which include mitochondrial fission-fusion dynamics, different post-translational modifications that fine-tune the process and recruitment of autophagy-related adaptor proteins on OMM. Mitochondrial membrane dynamics closely orchestrate the mitophagy pathway by either facilitating fission mediated separation of the damaged part or re-fusing the impaired mitochondria to the healthy pool to dilute out the impact of dysfunction. Mitochondrial damage initiates a series of events resulting in the localization of several proteins onto OMM. The recruitment of these proteins is very context-dependent and is also regulated by different post-translational modifications. There are only a few pieces of evidence wherein late-stage regulators such as SNARE and Rab proteins of the mitophagy pathway have been identified. Therefore, more studies characterising the functional players involved in mitophagosome expansion and fusion is required. Although well explored, the mitophagy pathway is surrounded with several controversies. Mitochondrial fission has been believed to precede mitophagy, but recent studies on MiD51, DRP1 and STX17 suggest otherwise. There are contrasting reports with respect to mitochondrial fission-fusion dynamics and mitophagy, and therefore careful spatio-temporal characterisation of proteins is required. As mitophagy is a predominant quality control pathway for maintaining mitochondrial homeostasis, any dysfunction in this highly orchestrated pathway leads to a myriad of disease conditions. Neurodegeneration is one such disease, and recent studies investigating the molecular details of mitophagy dysregulation in these disorders shed light into the mechanistic aspects of the disease pathology. However, extensive studies probing the mechanism of mitophagy dysregulation in neurodegenerative disorders is required to address the contradictory results and for better understanding of this pathway. The research outcome from such studies will help provide new insights for developing novel therapeutic strategies to ameliorate these debilitating diseases.
